# RPG interacts with E3-ligase CERBERUS to mediate rhizobial infection in *Lotus japonicus*

**DOI:** 10.1371/journal.pgen.1010621

**Published:** 2023-02-03

**Authors:** Xiaolin Li, Miaoxia Liu, Min Cai, David Chiasson, Martin Groth, Anne B. Heckmann, Trevor L. Wang, Martin Parniske, J. Allan Downie, Fang Xie

**Affiliations:** 1 National Key Laboratory of Plant Molecular Genetics, CAS Center for Excellence in Molecular Plant Sciences, Institute of Plant Physiology and Ecology, Chinese Academy of Sciences, Shanghai, China; 2 University of the Chinese Academy of Sciences, Beijing, China; 3 Faculty of Biology, University of Munich, Großhaderner Straße 2–4, Planegg-Martinsried, Germany; 4 John Innes Centre, Norwich Research Park, Norwich, United Kingdom; Tsinghua University, CHINA

## Abstract

Symbiotic interactions between rhizobia and legumes result in the formation of root nodules, which fix nitrogen that can be used for plant growth. Rhizobia usually invade legume roots through a plant-made tunnel-like structure called an infection thread (IT). RPG (Rhizobium-directed polar growth) encodes a coiled-coil protein that has been identified in *Medicago truncatula* as required for root nodule infection, but the function of RPG remains poorly understood. In this study, we identified and characterized *RPG* in *Lotus japonicus* and determined that it is required for IT formation. *RPG* was induced by *Mesorhizobium loti* or purified Nodulation factor and displayed an infection-specific expression pattern. Nodule inception (NIN) bound to the *RPG* promoter and induced its expression. We showed that RPG displayed punctate subcellular localization in *L*. *japonicus* root protoplasts and in root hairs infected by *M*. *loti*. The N-terminal predicted C2 lipid-binding domain of RPG was not required for this subcellular localization or for function. CERBERUS, a U-box E3 ligase which is also required for rhizobial infection, was found to be localized similarly in puncta. RPG co-localized and directly interacted with CERBERUS in the early endosome (TGN/EE) compartment and near the nuclei in root hairs after rhizobial inoculation. Our study sheds light on an RPG-CERBERUS protein complex that is involved in an exocytotic pathway mediating IT elongation.

## Author summary

In legume-rhizobium symbiotic interactions, growth of the plant-made infection thread (IT) through epidermal and cortical root cells is led by nuclear movement. RPG is a gene that is present in nitrogen-fixing root nodule (NFN) clades and is specifically required for IT formation. In this study, we found that RPG and CERBERUS co-localize and interact in root hairs close to the nucleus after rhizobial inoculation. Both are essential for normal IT growth, both co-localize in the TGN/EE compartment and are required for endomembrane dynamics during IT formation. Our study sheds light on how the RPG-CERBERUS complex promotes IT elongation.

## Introduction

Nitrogen-fixing root nodule symbioses (RNS) between legumes and their rhizobia symbionts are important because plants can obtain nitrogen from gaseous N_2_ that is reduced to NH_3_ by the rhizobia. The establishment and maintenance of this symbiosis depend on a molecular dialogue between the partners. The formation of N_2_-fixing nodules requires two developmental processes: nodule organogenesis and bacterial infection. Although the two processes can be genetically separated, they must be spatially and temporally coordinated to ensure nodule organogenesis at sites of bacterial infection [1]. In response to flavonoids exuded by the plant, rhizobia secrete decorated lipochito-oligosaccharide molecules called nodulation factors (NFs) that can activate nodule organogenesis, and can induce cellular changes associated with the initiation of bacterial infection [2].

In about 75% of the investigated legume-rhizobium symbioses, rhizobia invade legume roots *via* root hair intracellular infection [3]. Rhizobia attach to root hairs, triggering root-hair curling that entraps rhizobia and induces localized cell-wall degradation and rearrangement of the plant cytoskeleton, contributing to the formation of plant-made tunnel-like structures called the infection threads (ITs) [4]. Rhizobia colonize the ITs which grow through root cells, ultimately reaching the nodule primordium. The bacteria are then budded off surrounded by a plant-made membrane into plant cells in which they fix nitrogen using carbon supplied by the plant [5]. Genetic studies in *Lotus japonicus* and *Medicago truncatula* have identified several genes required for IT initiation and formation. Some are associated with changes in the actin cytoskeleton to promote IT growth. For example, PIR1 (121F-specific p53 inducible RNA 1), NAP1 (Nck-associated protein 1), and SCARN (SCAR-Nodulation) [6–8] are components of an actin assembly SCAR/WAVE complex, and ARPC1 (actin related protein complex 1) is a predicted subunit of the actin-related protein complex ARP2/3 [9]. Another component is a legume-specific pectate lyase, NPL, which may be involved in cell wall remodeling during IT initiation [10]. Rhizobia induce expression of root-hair-specific genes such as *CBS1* (Cystathionine-β-synthase-like 1), *RPG* (Rhizobium-directed polar growth), and *RINRK1* (Rhizobia infection receptor-like kinase 1) [11–13]. Although these genes have been identified, their biological functions in IT formation are not yet clear.

In response to rhizobia-secreted NFs, the root hair tips deform and entrap the bacteria; root hair cell nuclei then move to a location close to root hair tips and the plasma membrane invaginates to form an IT [4]. Subsequent IT progression within root hairs follows the path of the moving nucleus, regardless of its direction, supporting the idea that nuclear movement is necessary for IT guidance [14]. The Linker of Nucleoskeleton and Cytoskeleton (LINC) complex in *M*. *truncatula* is necessary for proper nuclear shaping and movement in *Medicago* root hairs, and it plays a role in IT initiation and nodulation [15]. A cytoplasmic column, rich in secretory organelles, accumulates between the IT and the nucleus [16] and encompasses a structure referred to as an infectosome [17,18]. In *L*. *japonicus*, the NF receptor NFR5 interacts with LjROP6 (Rho of Plants 6), which is activated by LjSPIKE1 (SPK1), a DOCK family GEF (guanine nucleotide exchange factor). LjSPK1-LjROP6 then guides polarized IT growth in root hairs [19,20]. *L*. *japonicus* CERBERUS and its orthologue LIN (Lumpy infection) in *M*. *truncatula* display punctate localization and interact with VAPYRIN, a protein of unknown function, to mediate IT polar growth [17,21,22]. Exo70H4, an exocyst subunit, co-localizes with VAPYRIN and LIN during rhizobia infection, suggesting that LIN, VAPYRIN and Exo70H4 may form symbiosis-specific machinery to regulate polar growth of IT [17].

NIN (Nodule inception) and RPG have been identified as two key genes which have been lost in most non-nodulating species but are essential for root nodule symbioses in nitrogen-fixing root nodule (NFN) clades including Fabales, Fagales, Cucurbitales, Rosales and Parasponia [23,24]. A *M*. *truncatula rpg* mutant formed abnormally thick and slow-growing ITs, indicating that RPG plays an important role in IT tip growth [11]. However, RPG has not been characterized in other legumes, and its precise biological function has remained elusive. In this study, we identified the *RPG* gene in *L*. *japonicus* and showed that it was required for IT formation. *RPG* displayed an infection-specific expression pattern and was directly induced by NIN. RPG showed punctate subcellular localization, and could co-localize and interact with CERBERUS to mediate IT formation.

## Results

### Identification of the *L*. *japonicus RPG* gene in infection-deficient mutants

**Fig 1:**
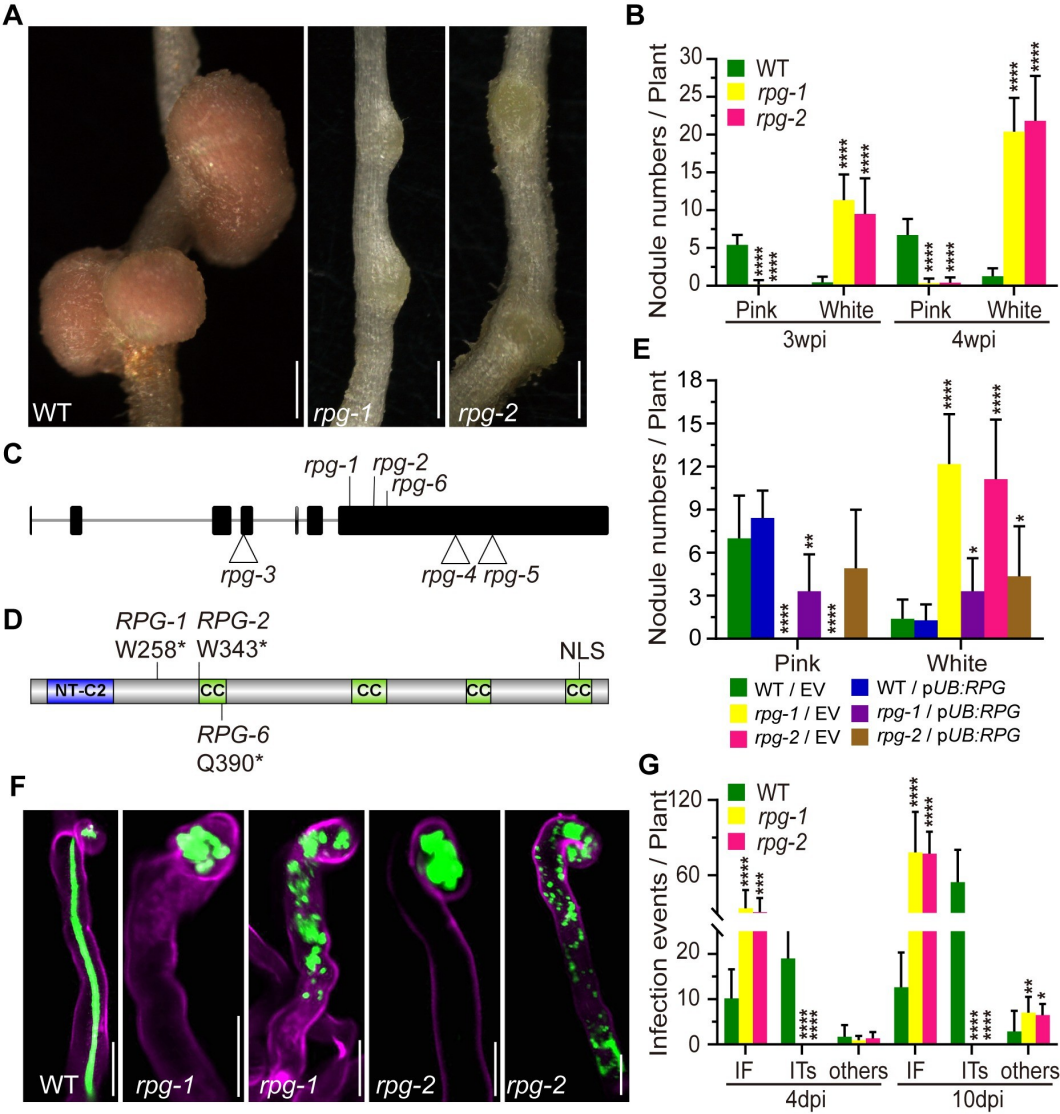
Phenotype and genotype of *rpg-1* and *rpg-2* mutants. (A-B) Nodule phenotype and the nodule numbers of wild type (WT) and *rpg* mutants two weeks after inoculation. The total number of nodules per plant was scored at three and four weeks after inoculation with *M*. *loti* R7A LacZ (n>10). (C) The gene structure of *RPG*, showing seven exons and six introns. The position of three EMS mutations, *rpg-1* (SL5706-3), *rpg-2* (SL454-2) and *rpg-6* (SL0181), and three *LORE1* insertion mutations, *rpg-3*, *rpg-4*, and *rpg-5* are shown. (D) Outline of RPG protein structure, indicating the predicted NT-C2 domain, four coiled-coil (CC) domains, and a predicted nuclear localization signal (NLS). The locations of translation stops (*) in *RPG-1*, *RPG-2* and *RPG-6* are indicated. Images (C and D) were created using IBS1.0.3 software. (E) The nodule numbers of WT, *rpg-1* and *rpg-2* mutant plants in roots transformed with the vector control (EV) or p*UB*:*RPG* and scored three weeks after inoculation with *M*. *loti* R7A/LacZ (n>7). (F) Infection phenotypes of WT, *rpg-1 and rpg-2* mutants were visualized by fluorescence microscopy of roots inoculated with *M*. *loti* R7A/GFP. Shown are a normal elongating IT in WT, and infection foci (IF) and abnormal ITs observed in *rpg-1 and rpg-2* mutants. Roots were scored seven days after inoculation and were counterstained with propidium iodide. Green fluorescence shows rhizobia and magenta fluorescence shows root hair stained with propidium iodide. (G) Number of infection events in WT plants and *rpg* mutants. The total number of infection events per plant was scored 4 and 10 days after inoculation with *M*. *loti* R7A/LacZ. IF, infection foci; ITs, infection threads; Others correspond to abnormal ITs in root hairs as illustrated in panel F (n>9). Asterisks indicate significant differences between WT and *rpg* mutants at the indicated time points (B and G), or between the EV control and experimental group (E) (Student’s *t*-test). Scale bars: 1 mm (A); 20 μm (F).

Two symbiosis-defective mutants (SL5706-3 and SL454-2) were isolated from an ethyl methanesulfonate (EMS) mutagenized population in *L*. *japonicus* Gifu B-129. Both mutant lines produced small white nodules three weeks after inoculation (Figs 1A, 1B and S1). SL5706-3 and SL454-2 were then crossed with the ecotype MG20 (Miyakojima) to generate mapping populations. F_1_ plants produced pink nodules two weeks after inoculation with *Mesorhizobium loti*. The nodulation phenotype was scored in F_2_ seedlings and revealed segregation of a monogenic recessive mutation in each line (SL5706-3: 145 nod^+^ and 37 nod^-^, χ^2^ value = 1.132; SL454-2: 237 nod^+^ and 60 nod^-^, χ^2^ value = 1.954). Rough mapping using established DNA markers (http://www.kazusa.or.jp/lotus/) revealed that both mutations were on *L*. *japonicus* linkage group 5, between markers TM0913 and TM0052. Two infection-related genes, *CERBERUS/LIN* and *RPG*, had previously been mapped to the corresponding region in *M*. *truncatula* [11,25,26]. We amplified and sequenced the genomic DNA corresponding to the coding region of *CERBERUS* and *RPG* in SL5706-3 and SL454-2. This revealed that neither line had a mutation in the *CERBERUS* gene, but each had a single point mutation in the putative ortholog of *RPG* (Lj5g3v1699100.1). SL5706-3 had a G to A transition at +3395 bp from the predicted start codon and SL454-2 had a G to A transition at +3650 bp; these mutations caused premature stops at residues W258 and W343 (Fig 1C and 1D and Table 1).

**Table 1:** *L*. *japonicus rpg* mutant alleles.

Allele (Previous name)	Mutation	Reading Frame Change
*rpg-1* (SL5706-3)	G3395A	W258 stop
*rpg-2* (SL454-2)	G3650A	W343 Stop
*rpg-3* (30053003)	LORE1 insertion in exon 4 (genome position 2381 bp)	Insertion of 7 aa after G122
*rpg-4* (30055099)	LORE1 insertion in exon 7 (genome position 4519 bp)	Insertion of 6 aa after N632
*rpg-5* (30010526)	LORE1 insertion in exon 7 (genome position 4911 bp)	Insertion of 36 aa after E763
*rpg-6* (SL0181)	C3778T	Q390 Stop

To test if these mutations caused infection defects in the two mutant lines, wild type (WT) cDNA was amplified from Gifu mRNA and inserted into a plasmid under the control of the *L*. *japonicus* ubiquitin promoter. This construct (p*UB*:*RPG*) was introduced into SL5706-3 and SL454-2 by *Agrobacterium rhizogenes*-mediated hairy-root transformation, restoring normal nodulation in both mutants (Figs 1E and S2). We conclude that the identified mutations in *RPG* caused the nodulation defect, and the alleles in SL5706-3 and SL454-2 were designated *rpg-1* and *rpg-2*, respectively.

*RPG* in *L*. *japonicus* is a 6.2-kb gene composed of seven exons separated by six introns (Fig 1C). Reverse transcription and DNA sequencing indicated that the *LjRPG* cDNA is 3528 bp, encoding a protein 1176 amino acids in length. The predicted protein was 60% identical to MtRPG. The LjRPG protein domain was analyzed by http://www.ch.embnet.org/software/COILS_form.html, which predicted that LjRPG has four long coiled-coil domains, similar to the MtRPG protein [11]. MtRPG has a nuclear localization signal (NLS) in its N-terminal domain [11]. However, with LjRPG the NLS was predicted (http://www.psort.org/) to be in its C-terminal domain (Fig 1D). As predicted for the *Parasponia* RPG [23,27], the LjRPG N-terminus had a predicted C2 (NT-C2) domain which is predicted to mediate lipid-binding (Fig 1D).

### Mutation of *RPG* blocks IT formation but not induction of early nodulation genes

Infection and nodulation phenotypes of the *rpg-1* and *rpg-2* mutants were analyzed after inoculation with *M*. *loti* R7A containing either a constitutively-expressed green fluorescent protein (GFP) or β-galactosidase (*lacZ*) marker gene. The WT plants produced elongated infection threads four days after inoculation (Fig 1F), but most infection events in the *rpg* mutants were blocked at the stage of formation of infection foci (Fig 1F). Some *rpg* root hairs contained bacteria but not associated normal IT were observed; these events were designated as “others” (Fig 1F). Such events have been observed in several other infection-defective mutants [6,7,13,28]. Analysis of infection events in the *rpg* mutants four and ten days post inoculation (dpi) revealed that most infection events were arrested as infection foci, and neither mutant formed any normal-looking infections until 10 dpi (Fig 1G).

Three *LORE1* insertion mutants [29,30] were obtained for *rpg* and the alleles were designated *rpg-3*, *rpg-4*, and *rpg-5* (Fig 1C and Table 1). A third EMS-induced mutant line with an infection defect (SL0181) was also isolated and the mutation mapped in a similar manner to *rpg-1* and *rpg-2* (S3A and S3B Fig). SL0181 was found to have a C3778T transition in the sequence of *RPG* leading to a premature stop codon at Q390, then it was designated *rpg-6* (Figs 1C and 1D and S3C). Similar to the *rpg-1* and *rpg-2* mutants, most rhizobial infections in the *LORE1* insertion mutants did not go further than infection foci, although some infection threads were observed (S4A–S4C Fig). However, the *LORE1 rpg* mutants formed pink nodules three weeks after inoculation. The *rpg-3* mutant produced a similar number of mature-looking pink nodules as the WT, whereas *rpg-4* and *rpg-5* had fewer pink nodules (S4B, S4D and S4E Fig). The *rpg-6* mutant had a strongly reduced number of nodules and a high number of uninfected nodule primordia (S5A and S5B Fig). *RPG* expression was measured by quantitative reverse transcription (qRT)-PCR in five *rpg* mutants. *RPG* transcript levels were significantly decreased in *rpg-1* and *rpg-2* but not in *rpg-3* or *rpg-4* mutants (S6 Fig).

*NIN*, *NPL*, *RINRK1* and *VPY1* are all induced by rhizobial infection [10,13,22,31]. These genes were all expressed at similar levels in the *rpg-1* and *rpg-2* mutants as in the WT (S7 Fig) indicating that *RPG* is not required for their induction by rhizobia.

Arbuscular mycorrhization by *Rhizofagus irregularis* was also scored in the *rpg-1* and *rpg-2* mutants; microscopic examination and quantification of infections five weeks after inoculation identified no difference in hyphal penetration, or arbuscule formation compared with WT (S8 Fig) indicating that *RPG* is required for infection by rhizobia but not by arbuscular mycorrhizal fungi (AMF).

### *RPG* is induced by NIN and shows infection-specific expression

**Fig 2:**
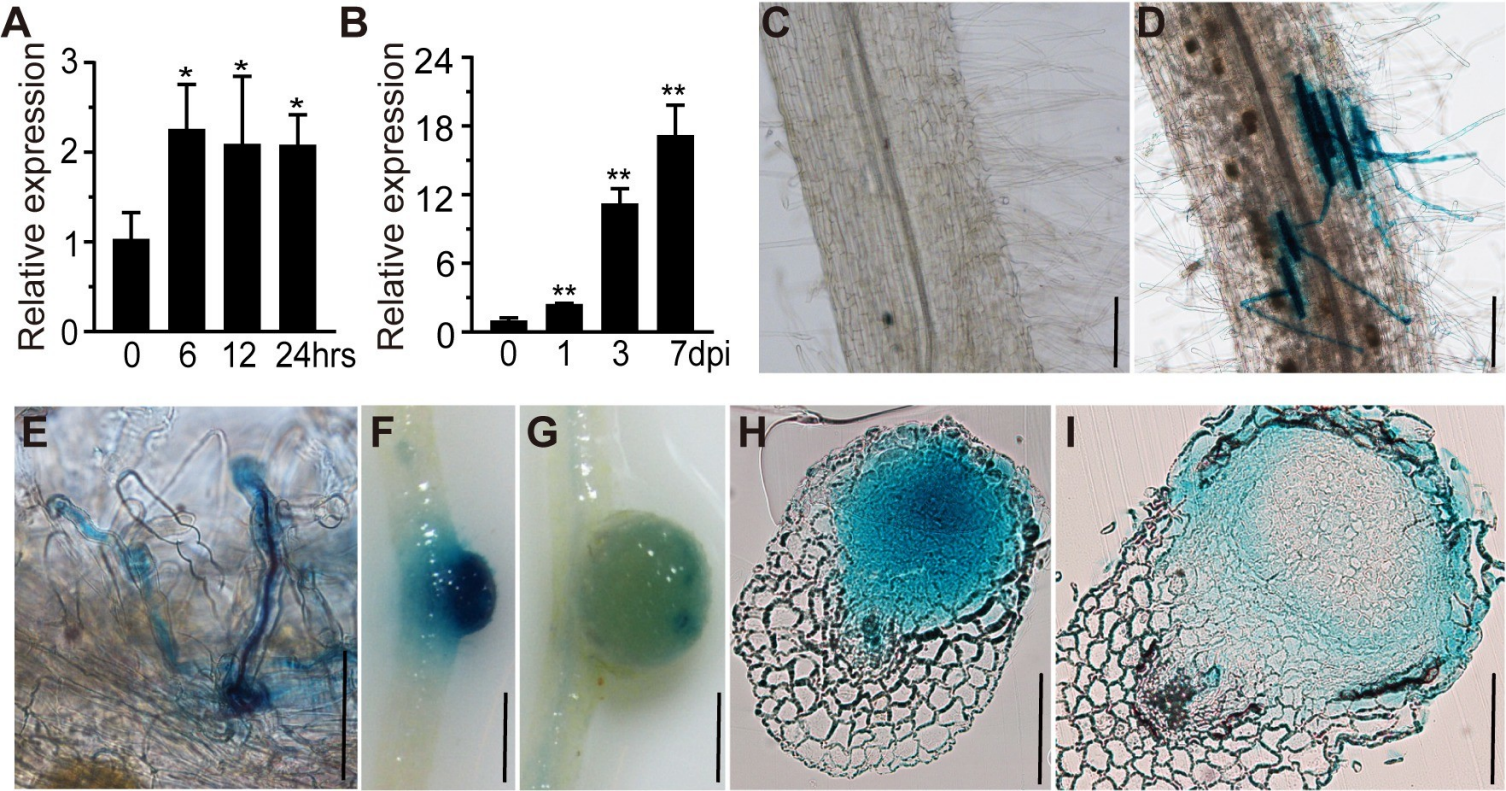
*RPG* expression pattern in *L*. *japonicus* roots. (A-B) qRT-PCR analysis of *RPG* transcript levels in roots of wild type (WT) *L*. *japonicus*. Samples were collected at 0, 6, 12, and 24 h after inoculation with purified Nod factor (A) or at 0, 1, 3 and 7 days after inoculation with *M*. *loti* R7A (B). Expression is relative to that of mock-treated samples (0 h or 0 dpi) and normalized to *L*. *japonicus Ubiquitin*. Asterisks indicate significant differences between the mock and the rhizobial/Nod factor treatments at the indicated time points by Students *t*-test. (C-G) p*RPG*:*GUS* expression patterns in *L*. *japonicus* roots and nodules. The constructs were expressed in wild type and the transgenic roots were stained with X-Gluc (blue). No GUS was detected in the absence of rhizobia inoculation (C). Strong GUS staining was detected in epidermal cells (D) and (E) and young nodules (F), but there was much lower staining in mature nodules (G). Bacteria were stained by magenta (purple) to indicate ITs (E). (H-I) Nodule sections showed that *pRPG*:*GUS* expressed in all cell layers of young nodules (H), but was only expressed in epidermal and nodule parenchyma cells in mature nodules (I). Scale bars: 100 μm (C-E and H-I); 1 mm (F-G).

*RPG* transcript levels were increased in roots at several time points after inoculation with *M*. *loti* or after addition of purified *M*. *loti* NF (Fig 2A and 2B). To investigate the spatial and temporal expression pattern of *RPG* during infection and nodulation, we used *A*. *rhizogenes* to transform *L*. *japonicus* WT roots with p*RPG*:*GUS*, which carries the *β-glucuronidase* (GUS) gene behind the *RPG* promoter. No GUS expression could be detected in un-inoculated transformed roots (Fig 2C). *RPG* expression could be detected in both uninfected and infected root hairs; in the latter GUS activity co-localized with *lacZ*-marked *M*. *loti* around the infection zone three to five days after inoculation (Fig 2D and 2E). Strong GUS staining was observed in nodule primordia, but there was much less staining in mature nodules (Fig 2F and 2G). Sections of developing nodule primordia (at 5 dpi) revealed GUS expression in all cell layers (Fig 2H), although GUS expression was then restricted to the nodule parenchyma cells in mature nodules (14 dpi) (Fig 2I).

**Fig 3:**
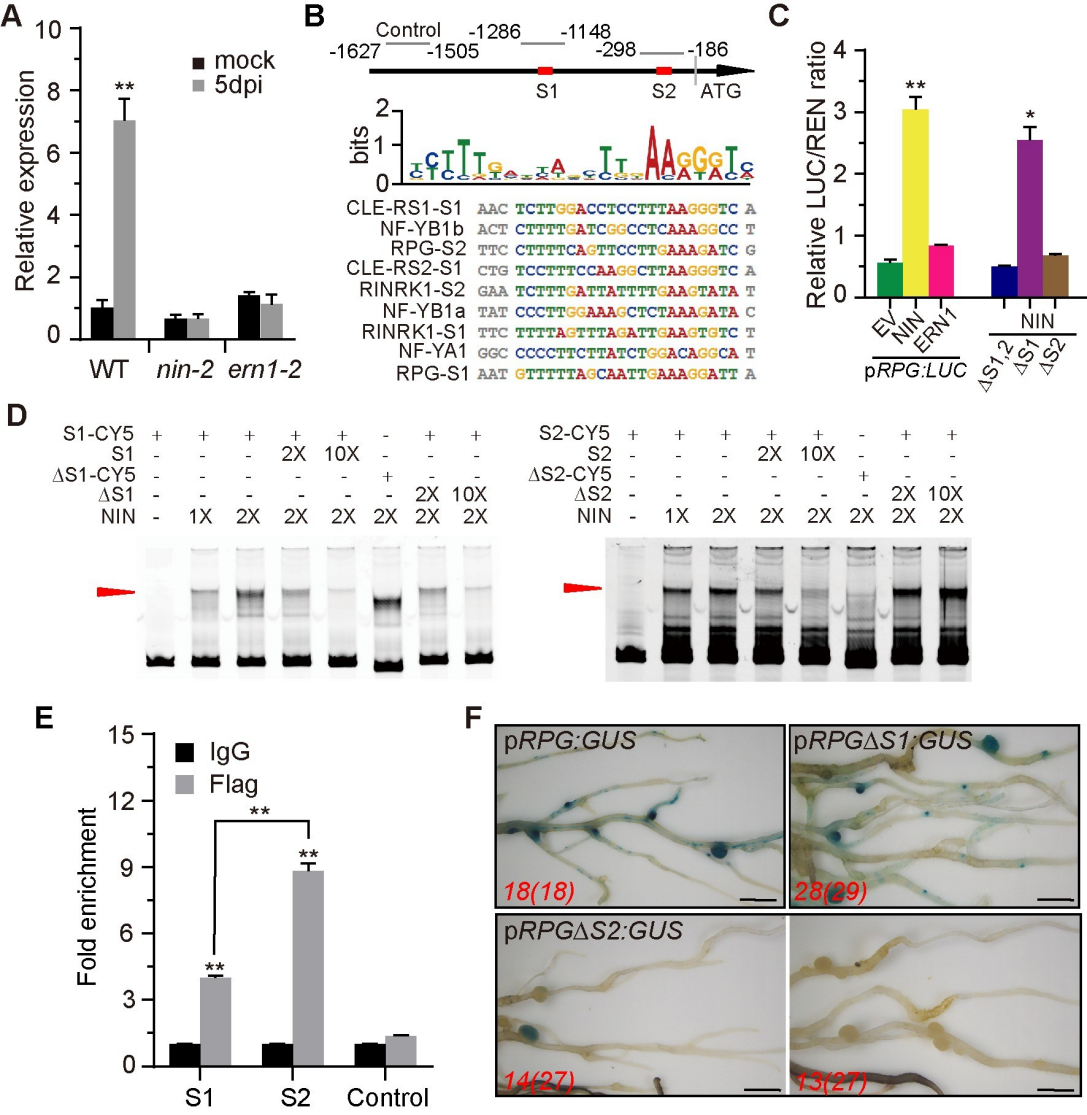
NIN induces *RPG* expression. (A) qRT-PCR analysis of *RPG* transcript levels in WT, *nin-2*, or *ern1-2* roots 5 days after inoculation with *M*. *loti* R7A. Expression is relative to that of mock-inoculated WT and normalized to *L*. *japonicus Ubiquitin*. Asterisks indicate significant differences between the mock-inoculated WT and the mutants (Students *t*-test). (B) Diagram of the *RPG* promoter region used for ChIP-qPCR analyses showing the locations of the putative NIN binding sites (NBS), S1 (-1157 bp) and S2 (-241 bp) upstream of the start codon. The sequence alignment shows the putative NBS of *RPG* aligned with previously identified NBS in *L*. *japonicus CLE-RS1*, *CLE-RS2*, *NF-YB1*, *NF-YA1*, and *RINRK1*. Images were created with the MEME suite. The relation ship between binding site and ChIP-qPCR primer region is illustrated by the numbered nucleotides at the top of the alignment. (C) The luciferase activity of co-expressed ERN1 or NIN with p*RPG*:LUX in *N*. *benthamiana* leaves. *Renilla* Luciferase (REN) activity was used to normalize for the efficiency of transformation. Asterisks indicate significant differences between NIN/ERN expressing constructs and an empty vector (EV) by Students *t*-test. (D) Gel-shift assays of NIN binding to the promoter of *RPG*. For S1 and S2, a 2-fold and 10-fold excess of unlabeled DNA fragments were added as competitors for binding. (E) Chip-qPCR analysis of NIN binding to the *RPG* promoter in *L*. *japonicus*. *pUb-NIN-Flag* was expressed in *L*. *japonicus* hairy roots 5 days after rhizobium inoculation using either an anti-Flag antibody or IgG as a negative control. The fold enrichment of NIN binding was determined relative to IgG (control) IPs. One representative biological replicate out of three is shown. Asterisks indicate significant differences between the anti-FLAG antibody and IgG (Students *t*-test). (F) Assays of p*RPG*:*GUS*, p*RPGΔS1*:*GUS*, p*RPGΔS2*:*GUS* expression in transformed *L*. *japonicus* roots. GUS activity was similar in p*RPG*:*GUS* and p*RPGΔS1*:*GUS*, but showed reduced levels in p*RPGΔS2*:*GUS*. Scale bars: 5 mm (F).

To analyze how *RPG* expression is regulated by NF signaling, we measured *RPG* expression in *nin-2* and *ern1-2* mutants. This revealed that *M*. *loti*-induced *RPG* expression requires *NIN* and *ERN1* in *L*. *japonicus* roots (Fig 3A). Two putative NIN-binding nucleotide sequences (NBS) [32] were identified at 1157 bp (S1) and 241 bp (S2) upstream of the *RPG* translation start codon (Fig 3B), but no ERN1-binding site [33] was found in the *RPG* promoter region. We used a dual-luciferase (dual-LUC) reporter assay to analyze whether NIN or ERN1 directly affects *RPG* transcription by co-expressing p*RPG*:*LUC* with p*35S*:*NIN* or p*35S*:*ERN1* in *Nicotiana benthamiana* leaf cells. Luciferase activity was quantified in leaf discs, revealing that NIN, but not ERN1, could induce *RPG* expression (Fig 3C left-hand panel). This was consistent with the fact that the *RPG* promoter region contains NIN-binding sites but not ERN1-binding sites. We then used an electrophoresis mobility shift assay (EMSA) to determine whether NIN could bind to these regions of the *RPG* promoter. A mobility shift was observed when the carboxyl-terminal half of the NIN recombinant protein was incubated with a synthetic oligonucleotide corresponding to the identified sequence in the *RPG* promoter S1 and S2 regions; an unlabeled competitor oligonucleotide outcompeted binding by the labelled probe (Fig 3D). Deletion of the conserved NBS of the S2 region (ΔS2, -29 bp) prevented NIN binding, but deletion of the S1 region (ΔS1, -35 bp) did not blocked NIN binding (Fig 3D). In a competition assay, unlabeled ΔS2 could not outcompete NIN binding to the labelled S2 region, whereas unlabeled ΔS1 could outcompete the NIN binding to the S1 probe (Fig 3D). These results all suggest that the S2 region is critical for NIN binding to the *RPG* promoter. To determine whether NIN could bind to the *RPG* promoter *in vivo*, chromatin immunoprecipitation (ChIP) was performed using NIN-FLAG-transformed *L*. *japonicus* hairy roots. A monoclonal antibody against FLAG was used for ChIP and an IgG antibody was used as a negative control. Primers for qPCR were designed to amplify different *RPG* fragments (Fig 3B). ChIP-qPCR results showed that both S1 and S2 could be enriched, but the amount of S2 enrichment is significantly higher than S1, while the control fragments were not (Fig 3E). To verify this, we used a dual-LUC system with p*RPG*:*LUC* containing deletions of S1 (p*RPGΔS1*:*LUC*), S2 (p*RPGΔS2*:*LUC*) or with both S1 and S2 deleted (p*RPGΔS1*,*2*:*LUC*) and co-expressed each with p*35S*:*NIN* in *N*. *benthamiana* leaves. The results showed that NIN could not induce expression of p*RPGΔS2*:*LUC* or p*RPGΔS1*,*2*:*LUC*, but could induce p*RPGΔS1*:*LUC* expression (Fig 3C right-hand panel). This indicated that S2 is essential for induction of *RPG* by NIN. The results were validated in *L*. *japonicus* by expressing p*RPG*:*GUS*, p*RPGΔS1*:*GUS*, or p*RPGΔS2*:*GUS* in transformed *L*. *japonicus* hairy roots; p*RPG*:*GUS* and p*RPG*ΔS1:*GUS* had similar expression patterns in roots inoculated with *M*. *loti* (Fig 3F). In contrast, about half of the p*RPG*Δ*S2*:*GUS* transgenic roots (13/27) had no detectable GUS expression and the remainder (14/27) showed weaker GUS staining than *pRPG*:*GUS* (Fig 3F). Based on these observations, we conclude that *RPG* is induced by NIN through an interaction with the S2 region, resulting in an infection-specific expression pattern.

### RPG displays punctate subcellular localization

**Fig 4:**
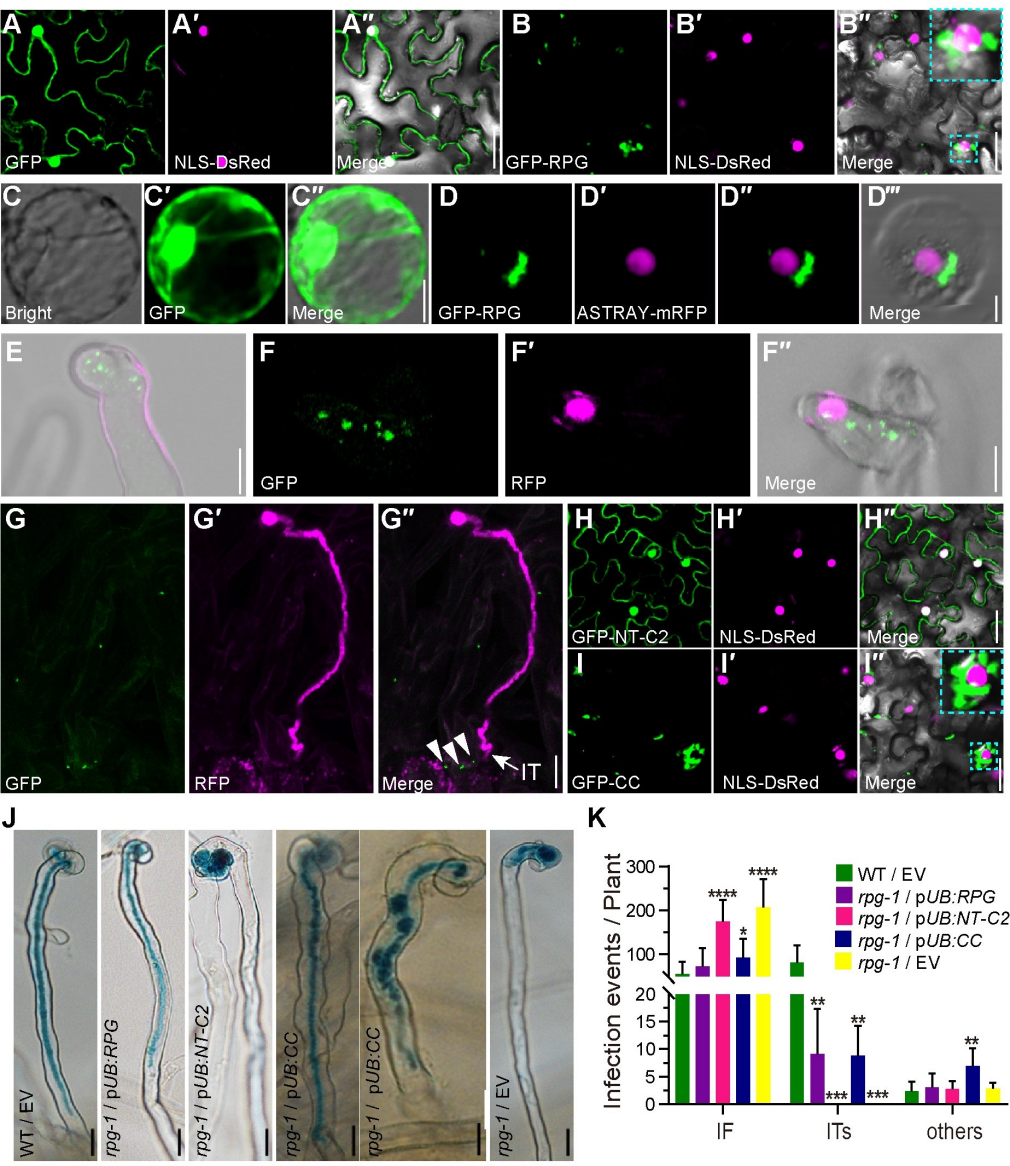
Subcellular localization of the RPG protein in *N*. *benthamiana* leaves and *L*. *japonicus* roots. (A, B, H and I) Confocal microscopy images of *N*. *benthamiana* leaves expressing p*35S*:*GFP* (A), p*35S*:*GFP-RPG* (B), and the separate RPG domains p*35S*:*GFP-NT-C2* (H) and p*35S*:*GFP-CC* (I). In each, the green, magenta and merged images are shown in adjacent panels. The nucleus was labeled with NLS-DsRed (magenta). Sections within an image that are outlined in dotted Cyan lines are showed enlarged in the top right corner of that image. (C -D) p*35S*:*GFP* (C) or p*35S*:*GFP-RPG* (green) and the nuclear marker, ASTRAY5-mRFP (magenta) (D), were co-expressed in *L*. *japonicus* root protoplasts using a DNA-PEG-calcium transfection method. (E to G) p*RPG*:*GFP-RPG* was introduced into *rpg-1* by *A*. *tumefaciens*-mediated stable transformation. RPG subcellular localization were analyzed using whole-mount immunolocalization with anti-GFP primary antibody and Alexa Fluor 488-conjugated Affinipure donkey anti-Mouse IgG secondary antibody, 5 or 10 days after inoculation with *M*. *loti* MAFF303099/RFP. Green shows RPG subcellular localization and magenta shows *M*. *loti*. Close arrowheads indicate RPG subcellular localization and arrow indicate IT (G). (J-K) Assays of complementation of the *rpg-1* mutant by the predicted NT-C2 domain (p*UB*:*NT-C2*) and by RPG lacking the NT-C2 domain (p*UB*:*CC*) showing the NT-C2 domain is not required for complementation of infection. Infection phenotypes (J) and Infection events (K) of the p*UB*:*NT-C2* or p*UB*:*CC* were expressed in *rpg-1*, and the phenotypes were scored after inoculation with *M*. *loti* R7A/LacZ (n>14). Asterisks indicate significant differences between the transformants with the *pUB-RPG* constructs and those with the empty vector (EV) (Students *t*-test). Scale bars: 25 μm (A-B and H-I); 10 μm (C-G); 20 μm (J).

To investigate the subcellular localization of RPG, we first used assays in *N*. *benthamiana* leaves. We had expected RPG to be localized to the nucleus based on prior results with *Medicago* RPG [11] and a predicted NLS at the C-terminus of LjRPG (Fig 1D). However, GFP-RPG made by fusing *GFP* with *RPG* cDNA and expressed by the 35S promoter showed strong fluorescence with punctate foci, some of which were close to the nucleus (Fig 4B). This observation was confirmed in *L*. *japonicus* root protoplasts in which *L*. *japonicus* ASTRAY, a homologue of *Arabidopsis thaliana* HY5 [34], was used as a nuclear marker. Co-expression of GFP-RPG and ASTRAY-mRFP in *L*. *japonicus* root protoplasts revealed puncta of GFP-RPG, some of which were close to, but distinct from the nucleoplasm (Fig 4D). As expected, expression of GFP alone (p*35S*:*GFP*) showed both nuclear and cytoplasmic localization (Fig 4A and 4C). Expressing the p*35S*:*GFP-RPG* construct in the *rpg-1* mutant rescued its infection defects and produced mature pink nodules (S9 Fig), revealing that this construct functioned normally in *L*. *japonicus*.

To analyze RPG subcellular localization in legumes after rhizobial inoculation, the *L*. *japonicus rpg-1* mutant was stably transformed with *GFP* fused to *RPG* cDNA downstream of the native *RPG* promoter (p*RPG*:*GFP-RPG*). Analysis of T_2_ plants of this transformant revealed that expression of p*RPG*:*GFP-RPG* in the *rpg-1* mutant resulted in formation of normal ITs and pink nodules as seen in the WT. In contrast, T_2_ segregants lacking p*RPG*:*GFP-RPG* (*rpg-1*) formed infection foci and white nodules as seen in the mutant (S10 Fig). This shows that the GFP-RPG fusion protein functioned in the transgenic roots of the *rpg-1* mutant. No GFP fluorescence could be reliably detected in live roots, so we immuno-localized the protein using GFP antiserum. There was little or no detectable signal in the absence of *M*. *loti*, but punctate localization of GFP-RPG protein was observed in root hairs (Fig 4E), with infection foci (Fig 4F) associated with growing ITs (Fig 4G) after inoculation with *M*. *loti* MAFF303099/RFP. Taken together, these results showed that RPG localizes in puncta when expressed in *N*. *benthamiana* leaves, *L*. *japonicus* root protoplasts, or in *L*. *japonicus* root hairs following inoculation with *M*. *loti*.

To analyze the domain of RPG that determines its subcellular localization, we made constructs in which the GFP was fused either to the RPG N-terminal NT-C2 domain contained in the first 300 amino acids of RPG (GFP-NT-C2) or the region of the protein (residues 170–1176) lacking the NT-C2 domain but containing all the C-terminal coiled-coil domains (GFP-CC). In *N*. *benthamiana* leaves GFP-NT-C2 was expressed in all cells (Fig 4H), similar to free GFP (Fig 4A), whereas GFP-CC displayed the same punctate localization as full-length GFP-RPG (Fig 4B and 4I); protein levels were quantified by immunoblotting with anti-GFP antiserum to confirm protein levels (S11 Fig). The observed localization suggested that the NT-C2 domain is not required for the observed subcellular localization of RPG. Constructs were then generated in which the NT-C2 or the protein lacking the NT-C2 domain were expressed by the *L*. *japonicus Ubiquitin* promoter [35]; these were expressed in roots of *rpg-1* using hairy root transformation. Expression of the *RPG* lacking the NT-C2 domain (p*UB*:*CC*) rescued the *rpg-1* infection defect as effectively as full-length *RPG* and the transformants formed normal ITs and pink mature nodules (Figs 4J, 4K and S12). No rescue was observed in roots expressing NT-C2 (p*UB*:*NT-C2*) (Figs 4J, 4K and S12) and in all cases transformation was confirmed with a separate GFP marker. Based on these data, we conclude that RPG displays punctate subcellular localization, and that the N-terminal C2 domain is not required for this subcellular localization or its biological function.

### RPG interacts with CERBERUS in yeast and *in planta*

**Fig 5:**
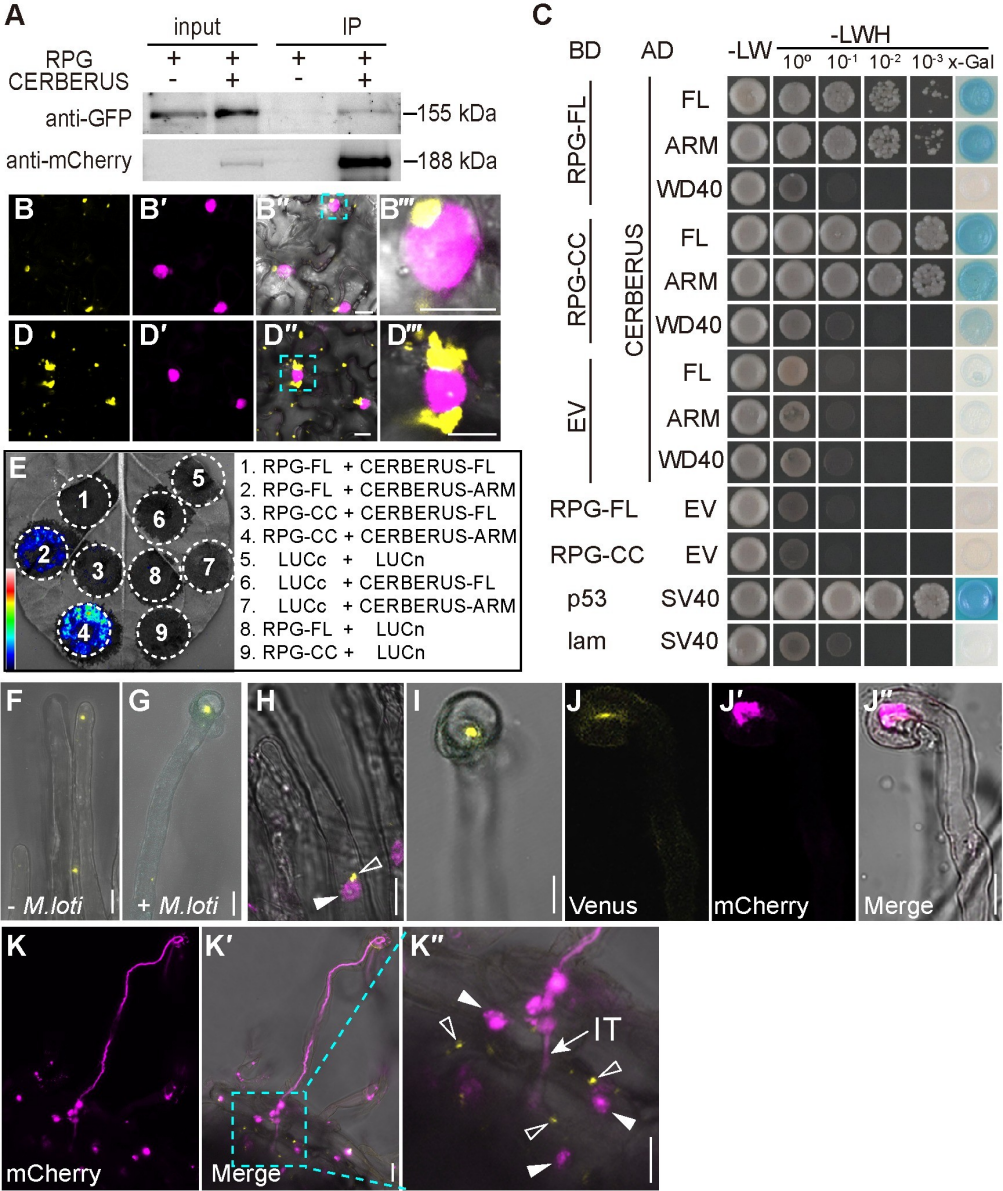
RPG interacts with CERBERUS. (A) Co-Immunoprecipitation (Co-IP) assay showing the interaction between GFP-RPG and CERBERUS-mCherry in *N*. *benthamiana* leaves. GFP-RPG and CERBERUS-mCherry were co-expressed in *N*. *benthamiana* leaves. Co-IP was assayed using anti-mCherry antibody, and the precipitated proteins were detected by immunoblot analysis with anti-mCherry and anti-GFP antibodies. One representative result out of two biological replicates is shown. (B and D) BiFC assays of full-length RPG and CERBERUS (B) or RPG-CC and CERBERUS-ARM (D) (yellow) and NLS-DsRed (magenta) in *N*. *benthamiana* leaves. The image shows strong Venus fluorescence localized in puncta, some of which were close to the nucleus. (B‴ and D‴) shows an enlargement of the area in outlined in cyan in the merged image (B″ and D″). (C) A GAL4-based yeast two-hybrid system was used to analyze the interaction between CERBERUS and full-length RPG (RPG-FL) or RPG lacking the NT-C2 domain (CC), and between CERBERUS ARM or WD40 and RPG-FL or RPG-CC. Potential interactions were assayed by growth on SD/-LWH (medium without histidine, leucine, or tryptophan) after gradient dilution. Images show the growth of co-transformants on selection media after three days. (E) Luciferase biomolecular complementation assays of the interaction between RPG and CERBERUS or RPG-CC and CERBERUS-ARM in *N*. *benthamiana* leaves. The indicated constructs were co-expressed in *N*. *benthamiana* leaves, and luciferase complementation imaging was conducted two days after agroinfiltration. LUCn, N-terminal fragment of firefly luciferase. LUCc, C-terminal fragment of firefly luciferase. Fluorescence signal intensity is indicated. (F-G) Live cell confocal images of RPG-CERBERUS BiFC construct was expressed in *L*. *japonicus* hairy roots. Venus fluorescence (yellow) was detected in *L*. *japonicus* root hairs before rhizobia inoculation (F), and in curled root hairs after rhizobia inoculation (G). (H-K) a RPG-CERBERUS BiFC construct was expressed in *M*. *truncatula sunn-1* by hairy root transformation. The transgenic roots were observed seven or ten days after inoculation with Sm1021/mCherry. mCherry (magenta) shows the nucleus (H) or Sm1021/mCherry (J, K). Close arrowheads indicate nucleus; Open arrowheads indicate Venus fluorescence produced by RPG interacting with CERBERUS; IT: Infection threads. Scale bars: 25 μm (C-D); 10 μm (F-J); 50 μm (K).

The punctate localization of RPG is similar to that reported for MtLIN and LjCERBERUS [17,22]. We hypothesized that RPG and CERBERUS may function together to promote IT formation. Indeed, co-expressed GFP-RPG and CERBERUS-mCherry in *N*. *benthamiana* leaves or *L*. *japonicus* root protoplasts showed punctate co-localization (S13 Fig). We then examined their interaction using co-immunoprecipitation (Co-IP) and bimolecular fluorescence complementation (BiFC) assays in *N*. *benthamiana* leaves. Co-IP assay showed that GFP-RPG was co-immunoprecipitated with CERBERUS-mCherry *in planta* (Fig 5A). For BiFC assays, RPG and CERBERUS were fused to split-Venus respectively. Co-expression of nVenus-CERBERUS and cVenus-RPG in *N*. *benthamiana* leaves and resulted in strong Venus fluorescence in puncta, some of which were close to the nucleus, identified by nuclear localized DsRed (NLS-DsRed) (Fig 5B).

We further used multiple assays to check which domains of RPG and CERBERUS mediate their interaction. Yeast two-hybrid (Y2H) assays confirmed that RPG could interact with full length CERBERUS, and showed that the CERBERUS Armadillo-like domain (ARM) (but not the WD40 domain) and the RPG coiled-coil domain (CC) were sufficient for the interaction (Fig 5C). To confirm the CERBERUS ARM and RPG CC domains promote the interaction, RPG-CC and CERBERUS-ARM were fused to nVenus and cVenus and co-expressed in *N*. *benthamiana* leaf cells. Strong puncta of Venus fluorescence were observed, and some of them close to nucleus (Fig 5D), a similar pattern as seen with the full-length protein fusions. Moreover, split-luciferase complementation imaging assays in *N*. *benthamiana* leaves confirmed that the RPG-CC and CERBERUS ARM domains can strongly interact (Fig 5E).

We further validated the RPG–CERBERUS interaction using BiFC in legumes. Transformation of *L*. *japonicus* roots with p*AtUBI10*:*nVenus-CERBERUS* and *pLjUBI1*:*cVenus-RPG* was selected using NLS-DsRed as a marker of transformation. The transgenic roots showed punctate fluorescence in root hairs before rhizobial inoculation (Fig 5F), and puncta in curled root hairs after rhizobial inoculation (Fig 5G). To enhance sensitivity, the same BiFC construct was expressed in roots of the *M*. *truncatula sunn-1* mutant, which shows increased levels of gene expression due to lack of autoregulation of nodulation [36]. After inoculation with *Sinorhizobium meliloti* 1021, which carried an mCherry reporter, punctate Venus fluorescence was detected in root hairs close to the nucleus (Fig 5H) and in curled root hairs (Fig 5I). The fluorescence was co-localized with rhizobia in the curled root hair (Fig 5J), and in elongated ITs (Fig 5K). These puncta labelled with the interacting proteins were similar to those labelled by GFP-LIN in *M*. *truncatula* PITs [17]. Based on these results, we conclude that RPG interacts with CERBERUS in infected root hairs, and this interaction and localization to puncta requires the RPG C-terminal coiled-coil domain.

### RPG interacts with CERBERUS at the TGN/EE compartment

**Fig 6:**
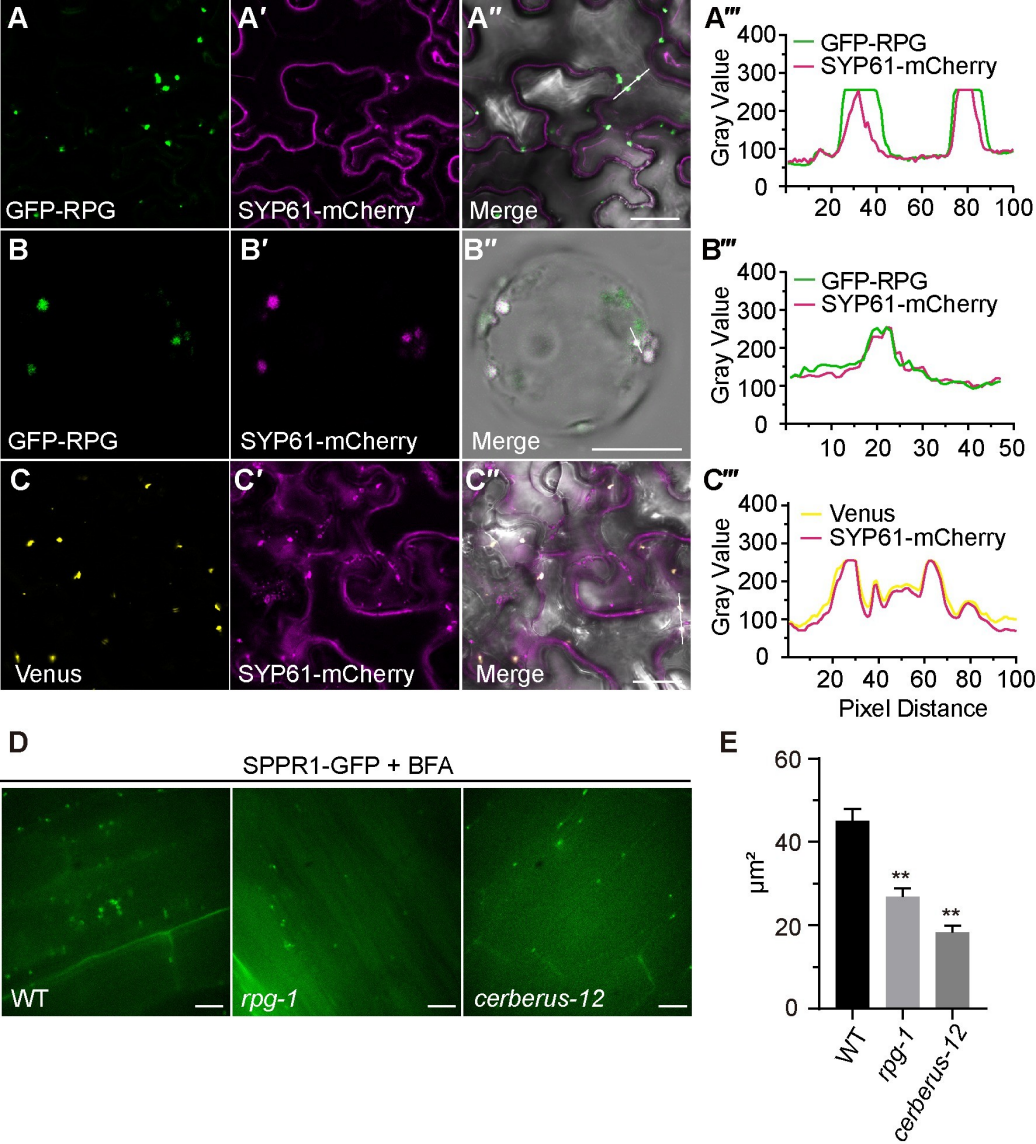
RPG interact CERBERUS at the TGN/EE compartment. (A-B) GFP-RPG (green) and TGN/EE marker SYP61-mCherry (magenta) were co-expressed in *N*. *benthamiana* leaf cells (A) or *L*. *japonicus* root protoplasts (B). (C) Co-expressed RPG-CERBERUS BiFC construct (yellow) and NLS-DsRed (magenta) in *N*. *benthamiana* leaf cells. Plots (A‴-C‴) show fluorescence intensities of the regions of interest (indicated by white line in [A″-C″]). (D) Confocal images of SPPR1-GFP in *L*. *japonicus* WT, *rpg-1* and *cerberus-12* transgenic roots inoculated with *M*. *loti* R7A/*lacZ* 2 days after Brefeldin-A induced BFA body formation. E, Quantification of BFA bodies size in WT, *rpg-1* and *cerberus-12* expressing SPPR1-GFP after 2 h BFA treatment. Asterisks indicate significant differences (Students *t*-test), between mutants and WT (error bars represent SE). Scale bars: 25 μm (A, C); 20 μm (B); 200 μm (D).

CERBERUS co-localizes with the TGN/EE compartment [22]. Hence, we tested whether RPG is also localized to this compartment. We co-expressed GFP-RPG or mCherry-RPG with the following subcellular markers in *N*. *benthamiana* leaves: Sec12-PHB-mCherry for the endoplasmic reticulum (ER); HAP3-GFP or SYP61-mCherry for the TGN/EE; ARA6-mCherry or mRFP-VSR2 for multivesicular bodies (MVB); CD3-963-GFP for the Golgi [37–41]. The results showed that RPG co-localized with ER and TGN/EE markers (Figs 6A, S14A and S14B) but not with the Golgi or MVB markers (S14C–S14E Fig). RPG subcellular localization was also analyzed in *L*. *japonicus* root protoplasts, in which GFP-RPG showed similar puncta and co-localized with the TGN marker SYP61 (Fig 6B). Co-expression of a BiFC construct (containing p*AtUBI10*:*nVenus-CERBERUS* and p*LjUBI1*:*cVenus-RPG*) with markers for the ER (Sec12-PHB-mCherry) and TGN/EE (SYP61-mCherry) in *N*. *benthamiana* leaves confirmed that the RPG-CERBERUS complex co-localized with ER and TGN/EE markers (Figs 6C and S14F).

The role of many TGN/EE-associated proteins is to regulate protein trafficking processes, such as vacuolar protein transport, endocytosis, and autophagy [42]. To test whether RPG and CERBERUS play a role in endomembrane trafficking pathways, we expressed a GFP secretion reporter [43] in roots of *rpg-1 and cerberus-12* mutants. We analyzed brefeldin-A (BFA)-induced BFA-body formation in the outer cortex of *rpg-1 and cerberus-12* root elongation zones over time using a GFP secretion reporter system [43]. These analyses revealed similar accumulation of GFP-labeled endomembrane compartments into BFA bodies in all three lines (Fig 6D) but it was delayed in *rpg-1 and cerberus-12*, resulting in the formation of smaller BFA bodies (Fig 6E). These results suggest that the RPG and CERBERUS may interact to function in endosome trafficking during IT formation.

## Discussion

RPG was identified as a conserved gene in nodulating FaFaCuRo clades [23,24], is required for IT polar growth in *M*. *truncatula* [11], and plays more important roles in root hair ITs than transcellular ITs in *L*. *japonicus* [44]. In this study, we identified 6 alleles of *RPG* in *L*. *japonicus*. All these 6 *rpg* mutants produced more infection foci, which mostly did not progress ITs; this resulted in no or few ITs, indicating that *RPG* is required for infection thread development. Mutants carrying either of two premature stop alleles, *rpg-1* and *rpg-2* did not induce ITs in response to *M*. *loti*. It was surprising therefore that, although mutants with *LORE1* insertions were greatly reduced in IT formation, they did form a few ITs 10 days after inoculation and some of these led to the formation of infected nodules, whereas at the same stage (2 wpi) the *rpg-1* and *rpg-2* mutants produced only small white uninfected bumps. We do not know why these LORE alleles have a weaker phenotype. Possibly the terminated forms of RPG predicted to be formed by *rpg-1* and *rpg-2* have negative effects. Alternatively, the gene products formed in the *rpg-4* and *rpg-5* mutants could retain some activity, or RNA processing allows the formation of a partially functional RPG.

*L*. *japonicus rpg* showed a similar symbiotic phenotype to other infection threads deficient (*itd*) mutants, such as *rinrk1*, *scarn*, *cerberus* and *npl* [7,10,13,25,45]. However, *RINRK1* was required for rhizobia-induced nodulation gene expression [13], although the induction of these genes by rhizobia was not affected in *scarn* and *rpg* mutants [7]. This suggests that the biological roles of SCARN and RPG in IT formation are different to RINRK1, which may involve the NF signaling transduction pathway.

*RPG* is induced by either purified NF or inoculation with *M*. *loti*. It was previously reported that *MtRPG* expression is dependent on NIN [46], and chromatin immunoprecipitation also showed that NIN could directly bind with the *RPG* promoter in *L*. *japonicus* roots [32]. We found that the induction of *RPG* by rhizobia requires both ERN1 and NIN, whereas NIN but not ERN1, could directly bind to the *RPG* promoter to activate *RPG* expression. This may be because ERN1 functions upstream of NIN, as has been suggested in *M*. *truncatula* [47]. Thus, ERN1 induction of *NIN* could induce *RPG* expression. Two predicted NIN binding sites were present in the *RPG* promoter, and although NIN could directly bind to both *in vitro*, but, only one was required for *RPG* induction in roots.

RPG contains a predicted NT-C2 domain and four predicted long coiled-coil domains. Proteins containing the NT-C2 domain, such as vertebrate estrogen early-induced gene1 (EEIG1) [48] and its ortholog in *Drosophila*, are required for uptake of dsRNA *via* the endocytic machinery to induce RNAi silencing [49]. In *Arabidopsis*, PLASTID MOVEMENT IMPAIRED 1 (PMI1) is a plant-specific C2-domain protein that is required for efficient movement of chloroplasts nuclei in response to light [50,51]. However, in this study, we did not observe a requirement for the predicted NT-C2 domain for IT formation suggesting that the lipid binding activity of RPG NT-C2 domain should be examined further. The RPG C-terminal long coiled-coil domain is sufficient for its protein subcellular localization and biological function. Long coiled-coils are highly versatile protein folding motifs that are involved in organelle architecture, in nuclear organization and with cytoskeletal motor proteins [52,53]. MtRPG can interact with MtIEF (Infection-related epidermal factor), a legume-specific protein which contains a coiled-coil region and a DUF761 domain with unknown function [54]. In this study, we found that RPG was localized in puncta, some of which were close to the nucleus in *N*. *benthamiana* leaves and *L*. *japonicus* root protoplasts. A very recent study in *M*. *truncatula* showed that the IT tip-to-nucleus microtubule connectivity is perturbed in *rpg-1* [55]. Together, all these studies strongly indicated that RPG plays an important role in nuclear-led IT elongation. MtLIN (the orthologue of CERBERUS) is localized at PIT tips, and also localizes in puncta associated with the nucleus [17]. RPG and CERBERUS interact near the nucleus and TGN/EE compartment. In plants, the TGN/EE network acts as a central sorting hub to direct newly synthesized and endocytosed cargo to the cell surface or vacuole [50,42]. Expression of the endosomal reporter SPPR1-GFP in *rpg-1* and *cerberus-12* roots, revealed that BFA-body formation was delayed in *rpg-1* and *cerberus-12* mutant roots. This was similar with the SCAR/WAVE component MtAPI (LjSCARN orthologue in *M*. *truncatula*, which controls endomembrane trafficking to establish cell wall properties during IT formation [56]. Moreover, MtLIN co-localizes and interacts with an exocyst subunit, MtExo70H4, during rhizobial infection [17]. All these results, suggest that an RPG–CERBERUS complex could promote polar growth of ITs by affecting nuclear migration through a connection to endosome trafficking and/or cytoskeletal changes during IT formation.

CERBERUS contains a U-box domain and has auto-ubiquitination activity [22,25]. CERBERUS interacts with LjVPY1/2, but promotes LjVPY1/2 accumulation in *N*. *benthamiana* and *L*. *japonicus* [22]. Despite persistent efforts, we were unable to express and purify RPG from *Escherichia coli*, and RPG expression in *N*. *benthamiana* was too low to perform an *in vitro* ubiquitination assay for CERBERUS and RPG. Moreover, LIN-VPY-Exo70H4 form a protein complex in infectosome in *M*. *truncatula* root hairs [17]. It will be very important to determine in the future the detailed molecular mechanisms of how the RPG, CERBERUS, VAPRYIN, and exocyst polar growth machinery operates in IT formation.

## Materials and methods

### Plant materials and strains

The *L*. *japonicus* ecotypes Gifu B-129 and Myakojima (MG-20) and mutant lines *nin-2*, *ern1-2* [57], and *cerberus-12* [22] were used in this study. For *M*. *truncatula*, the mutant line *sunn-1* [58] was used. The mutant lines *rpg-1* (SL5706-3), *rpg-2* (SL454-2), and *rpg-6* (SL0181) were isolated from forward genetic screening of an EMS mutagenesis population of *L*. *japonicus* Gifu B-129. Other *rpg* allelles were obtained from a *LORE1* retrotransposon insertion mutagenesis pool [29]. The transposon insertion in each gene was verified by PCR product sequencing; primers are shown in S1 Table. *Meshorhizobium loti* R7A, constitutively expressing GFP or *lacZ* (referred to as R7A GFP or R7A LacZ), or *M*. *loti* MAFF303099 carrying RFP, or DsRED were used for *L*. *japonicus* nodulation experiments, and *Sinorhizobium meliloti* 1021-mCherry was used for *M*. *truncatula* nodulation experiments. Spores of the mycorrhizal fungus *Rhizophagus irregularis* were used for analysis of AM symbiotic phenotypes. For hairy root transformation of *L*. *japonicus* or *M*. *truncatula* roots, *Agrobacterium rhizogenes* strain AR1193 was used. *A*. *tumefaciens* strain EHA105 or GV3101 (pSoup) were used for *N*. *benthamiana* transient expression and stable transformation of *L*. *japonicus* as previously described [59]. Plasmids were cloned in *Escherichia coli* DH10B or DH5α. *Saccharomyces cerevisiae* strain AH109 was used for the yeast two-hybrid assay.

### Cloning, DNA manipulation, and plasmid construction

For genetic complementation, the coding sequence (CDS) of RPG was amplified from a cDNA library of inoculated Gifu roots using the primers *RPG-XbaI-F* and *RPG-AscI-R*. The PCR products and pUB-GFP plasmid were digested with *XbaI* and *AscI*, then RPG was inserted into pUB-GFP to form p*UB*:*RPG*. The NT-C2 and entire CC domains of RPG were amplified by PCR using the primers *RPG-attB-F/NT-C2-attB-R* or *CC-attB-F/RPG-attB-R*. The PCR product was inserted into pDONR207 *via* a BP reaction (Invitrogen, Waltham, MA, USA) and combined into pUB-GW-GFP to generate the p*UB*:*NT-C2* or p*UB*:*CC* construct *via* the LR reaction (Invitrogen).

For yeast two-hybrid assays, *RPG* PCR products were recombined into pDONR207 using the BP reaction. RPG/pDONR207, NT-C2/pDONR207, and CC/pDONR207 were recombined into pDEST-GBKT7 or pDEST-GADT7 using the LR reaction.

For split-luciferase complementation imaging assays, *RPG*, *CERBERUS*, the RPG CC domain and the CERBERUS ARM domain were inserted into the destination vectors 771-LUCn and 772-LUCc following *KpnI* and *SalI* digestion. To determine the subcellular localization of RPG in *N*. *benthamiana* leaves, RPG/pDONR207, NT-C2/pDONR207, and CC/pDONR207 were recombined into destination vector pK7WGF2-NLS-DsRed, which was modified from pK7WGF2. The kanamycin resistance gene of pK7WGF2 was replaced with a fragment of *NLS-DsRed* which was driven by the ubiquitin promoter.

For co-localization and BiFC analyses in *N*. *benthamiana* leaves, *L*. *japonicus* plants, and *M*. *truncatula* hairy roots, mCherry-RPG constructs (co-localization assays) or RPG and CERBERUS, RPG-CC and CERBERUS-ARM constructs (BiFC assays) were generated with Golden Gate cloning [60]. The RPG and CERBERUS CDS were synthesized in the level 0 vector pL0V-C-41264 (Shanghai Xitubio Biotechnology) to generate p*L0M*-C-*RPG* or p*L0M*-C-*CERBERUS*. The entire CC domains of RPG and CERBERUS ARM domain contains the U-box were amplified using p*L0M-C-RPG* or p*L0M-C-CERBERUS* as template. The PCR products and pL0V-C-41264 vector were digested with BpiI to generate p*L0M-RPG-CC* or p*L0M-CERBERUS-ARM*. p*L0M-C-RPG* and the EC15111 vector were digested with BsaI to generate mCherry-RPG as the level 1 construct. This level 1 mCherry-RPG was assembled into EC50507 (https://www.ensa.ac.uk/) to generate the level 2 construct mCherry-RPG binary vector. p*L0M-C-RPG* or p*L0M-RPG-CC* was assembled into EC10048 to generate cVenus-RPG or cVenus-RPG-CC, and p*L0M-C-CERBERUS* or p*L0M-CERBERUS-ARM* was assembled into EC10044 to generate nVenus-CERBERUS or nVenus-CERBERUS-ARM. Finally, these constructs were assembled into EC50507, adding p*35S*:*NLS-DsRed* or p*35S*:*GUS* as a transgenic marker, to generate the BiFC construct p*AtUBI10*:*nVenus-CERBERUS/*p*LjUBI1*:*cVenus-RPG* or p*AtUBI10*:*nVenus-CERBERUS-ARM/*p*LjUBI1*:*cVenus-RPG-CC*. For transient expression in *L*. *japonicus* root protoplasts, the *GFP-RPG* fragment was amplified using RPG/pK7WGF2-NLS-DsRed as a template. The PCR products and pA7-GFP were digested with SpeI and BamHI, then GFP-RPG was inserted into pA7-GFP to form pA7-GFP-RPG. *ASTRAY* cDNA was amplified from Gifu cDNA using the primers *ASTRAY-BamHI-F* /*ASTRAY-BamHI-R*. The PCR products and pA7-mRPF were digested with BamHI, then inserted into pA7-mRFP to generate ASTRAY-mRFP by homologous recombination methods (Vazyme). To obtain stably transformed plants, RPG/pDONR207 was recombined into pK7WGF2. The 35S promoter in RPG/pK7WGF2 was replaced by *RPG* promoter to generate p*RPG*:*GFP-RPG*.

For the dual-luciferase reporter assay in *N*. *benthamiana*, the *RPG* promoter fragments or those with NBSs deleted were amplified *via* PCR using the primers shown in S1 Table. Single or multiple PCR products were then inserted into the pGreen II vector using homologous recombination methods (Vazyme) following KpnI and HindIII/SpeI digestion to generate the p*RPG*:*LUC*, p*RPGΔS1*:*LUC*, p*RPGΔS2*:*LUC*, and p*RPGΔS1*,*2*:*LUC* constructs. The effector construct was generated by inserting NIN or ERN1 CDS into the pRI101 vector (containing the 35S promoter) using the KpnI/EcoRI restriction sites.

For expression analysis of RPG in *L*. *japonicus* hairy roots, *RPG* promoter fragments (1581 bp upstream of their respective start codons) were amplified from genomic DNA extracted from Gifu leaves. The *RPG* fragments with NBS S1 or S2 deleted from the promoter were amplified using p*RPGΔS1*:*LUC* or p*RPGΔS2*:*LUC* as a template. PCR products were cloned into pDONR207 using a BP reaction, and combined into pKGWFS7-NLS-DsRed to generate the p*RPG*:*GUS*, p*RPGΔS1*:*GUS* or p*RPGΔS2*:*GUS* constructs via LR reaction. The pKGWFS7.0-NLS-DsRed vector was modified from pKGWFS7.0 by replacing the kanamycin with the *ubiquitin drive NLS-DsRed*.

All PCR amplification was performed using MAX (Vazyme), and all constructs were confirmed by DNA sequencing. Primers are shown in S1 Table and constructs are listed in S2 Table.

### Map-based cloning

The *rpg-1*, *rpg-2* and *rpg-6* mutations were mapped using an F_2_ populations established by using SL5706-3, SL454-2 and SL0181 as pollen donors to *L*. *japonicus* ecotype MG20. Plants were inoculated with *M*. *loti* R7A LacZ and scored at 21 dpi for the nodulation phenotype. Genomic DNA was extracted from leaves as previously described [61]. Primer sequences and information for SSR markers were retrieved from the miyakogusa.jp website (http://www.kazusa.or.jp/lotus/).

### Plant growth conditions, symbiotic inoculations, and phenotype observation

*L*. *japonicus* or *M*. *truncatula* seeds were scarified, surface sterilized, and grown as previously described [7,62]. After five to seven days of growth, seedlings were inoculated with *M*. *loti* R7A LacZ, MAFF303099 GFP or DsRED strains. Nodule number was scored three and four weeks after inoculation. For phenotyping of *rpg-6*, WT and SL0181 (M4) plants were grown at 24°C, 16 hours photoperiod as previously described [63]. Nodules and uninfected nodule primordia were quantified 14 days after inoculation with DsRed-expressing *M*. *loti* MAFF303099 by fluorescence and bright-field microscopy (Leica MZ16 FA). The number of infection events was determined by microscopy of the whole root stained with 5-bromo-4-chloro-3-indolylbeta-D-galacto-pyranoside (X-Gal) at 4 and 10 dpi with *M*. *loti* R7A LacZ; at least nine plants were scored at each time point. LacZ staining, observation of GFP-marked *M*. *loti*-inoculated roots, and light microscopy of nodule sections were performed as previously described [7].

For mycorrhizal analysis, *L*. *japonicus* seedlings were grown in pots containing sand and perlite (1:4) with sterile *R*. *irregularis* spores. The roots were stained with ink/vinegar and fungal structures quantified five weeks after inoculation as previously described [64]. The samples were analyzed at 10x magnification with a bright-field microscope (Nikon Eclipse). Images of roots stained with WGA-Alexa Fluor 488 were taken with confocal microscopy (Olympus FV10-ASW).

### Complementation tests

Roots of WT, *rpg-1* or *rpg-2* mutants were transformed with p*UB*:*RPG* using *A*. *rhizogenes* AR1193-mediated hairy root transformation. The transformed chimeric plants were transplanted into vermiculite/perlite pots and inoculated with *M*. *loti* R7A LacZ after five to seven days. Infection events were analyzed at 7 dpi and the nodulation phenotypes were scored two or three weeks after inoculation.

### Gene expression pattern analysis

*L*. *japonicus* WT (Gifu), *rpg-1*, *rpg-2*, *nin-2*, and *ern1-2* seedlings were grown on FP agar medium for seven days. Plants were then either inoculated with *M*. *loti* R7A or 10 nM purified *M*. *loti* NFs was added. Samples were collected at 0, 1, 3, and 7 days after *M*. *loti* inoculation or 0, 6, 12, and 24 h after NF treatment, immediately frozen in liquid nitrogen, and stored at -80°C until use. Total RNA was extracted using the TRIpure Isolation Reagent (Aidlab, China); RNA was reverse transcribed using TransScript one-step gDNA removal and cDNA synthesis SuperMix (Trans Gen Biotech). qRT-PCR reactions were performed with the TOYOBO SYBR Green Realtime PCR Master Mix (TOYOBO) and analyzed with a step-one Plus PCR system (ABI). *Lotus* Ubiquitin (Lj5g3v2060710.1) was used as a reference gene to normalize expression. All of the primers used for qRT-PCR of target transcripts are shown in S1 Table.

For promoter GUS assays, the p*RPG*:*GUS*, p*RPGΔS1*:*GUS*, p*RPGΔS2*:*GUS* construct was transferred into AR1193, then expressed in *L*. *japonicus* WT (Gifu) by hairy root transformation. Transgenic plants were transferred into a 1:1 vermiculite:perlite mixture and inoculated with *M*. *loti* R7A LacZ after 5–7 days. GUS expression was analyzed at 7 and 14 dpi.

### Electrophoresis mobility shift assays (EMSA)

NIN carrying a C-terminal His tag was purified as previously described [10]. The RPG promoter regions S1(−1037 to −1231 bp), S2 (−141 to −339 bp), ΔS1, and ΔS2 were amplified *via* PCR using p*RPG*:*GUS*, p*RPGΔS1*:*GUS*, and p*RPGΔS2*:*GUS* as the template; primers are shown in S1 Table. PCR products were fluorescently labeled at the 5’ ends with Cy5 (Yingjun Corp. China) and purified by gel extraction (OMEGA Bio-TEK). Fluorescently labeled DNA was then detected using a Biophotometer Plus (Eppendorf) and 1 nM of DNA incubated with the purified NIN protein in 20 μL of binding buffer (20 mm Tris, pH 7.5; 5% [w/v] glycerol; 10 mm MgCl_2_; 0.25 mm dithiothreitol; 0.8 μg bovine serum albumin [BSA]; and 1 μg salmon sperm DNA). After incubation at 30°C for 20 min, the products were electrophoresed at 4°C on a 6% native polyacrylamide gel in Tris-borate/EDTA buffer for 2 h at 100 V. Fluorescence in the gel was detected with a Starion FLA-9000 (FujiFilm).

### Dual-luciferase reporter assays in *N*. *benthamiana*

The dual-luciferase reporter assay was performed in *N*. *benthamiana* leaves as previously described [62]. The indicated constructs were transferred into *A*. *tumefaciens* GV3101 (pSoup), then introduced into *N*. *benthamiana* leaves by infiltration. After two days, the LUC/REN ratio was measured with the dual-luciferase reporter assay system following the manufacturer’s instruction (Promega). Mean values and standard deviations were calculated from three biological replicates.

### Chromatin immunoprecipitation analysis

Chromatin immunoprecipitation (ChIP) was assayed using a Plant ChIP Kit (EPT-P-2014-24, EpiQuik). Briefly, 1g of transgenic hairy roots expressing p*UB*:*NIN-Flag-GR* was harvested 6h after the addition of dexamethasone (DEX) to induce *NIN* expression. The roots were then cross-linked by adding 20 mL 1% formaldehyde for 10 min under a vacuum. The crosslinking reaction was quenched with 2.5 mL 1M glycine solution. The roots were then frozen in liquid nitrogen and ground to a fine power, then the nuclei were isolated through two layers of Miracloth (475855-1R, Millipore). Cross-linked chromatin was sheared using a sonicator (UCD-200, Bioruptor) for 15s at 40% duty cycle, until most of the resulting DNA fragments are between 200 and 600 bp. Immunoprecipitation was performed using anti-FLAG antibody (HOA012FL01, HuiOu). qPCR analysis then was performed using the primers listed in S1 Table.

### Protein subcellular localization and co-localization in *N*. *benthamiana* leaves or *L*. *japonicus* root protoplasts

CERBERUS-mCherry and the other organelle markers used for protein subcellular localization analysis in *N*. *benthamiana* leaves have been described previously [22]. The constructs were introduced into *A*. *tumefaciens* EHA105 by electroporation, and *N*. *benthamiana* leaves were infiltrated with the resulting strains either alone or together. All were infiltrated with p19, which inhibits gene silencing [65]. Images were taken two days later with laser scanning confocal microscopy (Leica TCS SP8). The level of colocalization was analyzed using ImageJ. All protein subcellular localization assays were repeated at least three times.

For transient expression in *L*. *japonicus* root protoplasts, constructs (pA7-GFP-RPG, pA7-GFP-RPG, and ASTRAY*-*mRFP) were transiently expressed or co-expressed in *L*. *japonicus* root protoplasts using a DNA-PEG-calcium transfection method [66]. Images were taken 16 h after transfection by laser scanning confocal microscopy (Leica TCS SP8). For GFP, the filter sets for excitation and emission were 488 nm and 498–550 nm, respectively; for mCherry, DsRed, and mRFP, they were 561 nm and 575–650 nm. The level of colocalization was analyzed using ImageJ. All protein subcellular localization assays were repeated at least three times.

### Whole-mount immunolocalization assays for RPG subcellular localization in *L*. *japonicus* roots

The p*RPG*:*GFP-RPG* plasmid was introduced into *A*. *tumefaciens* strain EHA105, then expressed in *rpg-1* by *A*. *tumefaciens-*mediated transformation [59] to generate stably transformed plants.

RPG subcellular localization was analyzed using whole-mount immunolocalization as previously described [67]. Briefly, transgenic plants were inoculated with *M*. *loti* MAFF303099/RFP and 5–7 days after inoculation, the roots were submerged in fixative solution (4% formaldehyde in phosphate-buffered saline (PBS)) in a vacuum desiccator for 1 h. Fixative solution was removed and seedlings were washed two times for 5–10 min each with 1x PBS at room temperature. This was followed by two washes with water for 5 min each. Root pieces were transferred to microscope slides and dried overnight, then root tissue was rehydrated by pipetting 1**×** PBS onto the microscope slides and incubating for 5 min at room temperature. Roots were collected into 2 mL EP tubes, then permeated with 2% Driselase in PBS and incubated for 60 min at 37°C, followed by five washes with 1**×** PBS for 10 min each. A mixture of 3% IGEPAL CA-630 with 10% DMSO in PBS was added, then after 1 h the tissues were washed with 1**×** PBS five times for 10 min each. After blocking with 3% BSA in PBS, the fixed roots were incubated with primary antibody (anti-GFP, 1:300, Abmart) for 4 h at 37°C. Alexa Fluor 488-conjugated AffiniPure Donkey Anti-Mouse IgG secondary antibody (1:500, Jackson) was added and incubated for 3 h at 37°C, then samples were washed with 1**×** PBS five times for 10 min each. Images were taken with a confocal microscope (Leica TCS SP8). For RFP, the filter sets for excitation and emission were 561 nm and 575–650 nm, respectively; for Alexa 488, they were 488 nm and 498–519 nm.

### Protein-protein interaction assays

Interactions between RPG and CERBERUS were assayed using the yeast two- hybrid system as previously described [22]. The yeast strain AH109 was transformed with the constructs in destination vectors using lithium acetate transformation (Yeast Protocols Handbook PT3024-1, Clontech). The transformants were grown on synthetic defined medium (0.67% yeast nitrogen base, 2% Bacto-agar and amino acid mix) without the appropriate auxotrophic markers after gradient dilution. These assays were repeated three times.

For split-luciferase complementation imaging assays in *N*. *benthamiana* leaves,

LUCc-RPG was co-expressed with CERBERUS-LUCn, ARM-LUCn, in *N*. *benthamiana* leaves *via* agroinfiltration with p19, which inhibits gene silencing. The transformed plants were grown in a growth chamber. Two days after infiltration, images were captured by CCD (TANON 5200, China) after 1 mM luciferin (Promega) was sprayed onto the leaves. All images were acquired using the same exposure settings. Each interaction group was validated with three replicates, and two or three independent experiments were performed.

For co-immunoprecipitation (Co-IP) assays, GFP-RPG and CERBERUS-mCherry were co-expressed in *N*. *benthamiana* leaves. The samples were harvested 60 h after agroinfiltration, and approximately 0.6 g of plant tissue was extracted with 2 ml lysis buffer (50 mM Tris-MES at pH 8.0, 0.5 M sucrose, 1 mM MgCl_2_, 10 mM EDTA, 5 mM DTT, 0.2% NP-40, 1 mM phenylmethanesulfonyl fluoride (PMSF] and proteinase inhibitor cocktail tablet (Roche]) for 15 min then centrifuged at 12,000 rpm for 10 min. The supernatants were collected for Co-IP. Samples were incubated for 1.5 h with 30 μL Anti-RFP Affinity beads 4FF (Cat. SA072C, SMART) at 4°C on a rotating wheel, then centrifuged at 2000 rpm for 2 min at 4°C. The beads were washed and analyzed by immunoblotting using anti-mCherry (Cat.T0090, Affinity Biosciences, Cincinnati, OH, USA) and anti-GFP antibody (Cat. M20004L, Abmart). Approximately 10 μL of lysis buffer containing total protein was loaded as the input control.

For BiFC assays, the construct p*AtUBI10*:*nVenus-CERBERUS/*p*LjUBI1*:*cVenus-RPG* was expressed in *N*. *benthamiana* leaves by agroinfiltration with p19. Transformed plants were grown in a growth chamber, and images were captured two to three days later by laser scanning confocal microscopy (Leica TCS SP8). The BiFC construct was also expressed in *L*. *japonicus* Gifu or *M*. *truncatula sunn-1* by hairy root transformation. The transgenic hairy roots were scored based on the NLS-DsRed marker and inoculated with *M*. *loti* MAFF303099/RFP or Sm1021/mCherry (OD_600_: 0.001). Images were analyzed at 5–7 dpi. The filter sets for excitation and emission were 514 nm and 524 to 545 nm, respectively, 561 nm for Venus, and 600 to 630 nm for DsRed. All BiFC experiments were repeated twice, and five leaves or roots were analyzed each time.

### Analysis of endomembrane dynamics

Roots of *L*. *japonicus* WT, *rpg-1* and *cerberus-12* mutants were transformed with SPPR1-GFP reporter [43], the transformed roots were inoculated with *M*. *loti* R7A/*lacZ* 2 dpi and treated with 50 μM brefeldin A (BFA). The GFP fluorescence signal intensity and distribution were analyzed after 2h treatment. Images were taken by spinning disk confocal (ANDOR REVOLUTION XD). For image processing and analysis, the software ImageJ was used to count the area formed by BFA body.

### Statistical analysis

Statistical significance was analyzed by Student’s *t-test* (*P < 0.05, **P < 0.01, ***P < 0.001, ****P < 0.0001) and error bars indicate SD. Histograms were generated using GraphPad Prism 8.0 software.

## Supporting information

S1 Fig Sections of nodules formed on *L*. *japonicus rpg* mutants. (A-C) Section of 3-week old nodules on wild type plants were well colonization, but no colonization was observed in *rpg-1* and *rpg-2*. Scale bars: 100 μm.
(TIF)

S2 Fig *RPG* complementation of *rpg-1* and *rpg-2* mutants. Representative transgenic hairy roots of *L*. *japonicus* WT, *rpg-1* and *rpg-2* plants transformed with the empty vector control (EV) or p*UB*:*RPG* three weeks after inoculation with *M*. *loti* R7A/LacZ. The upper panels are epifluorescence microscopy images showing GFP expression from the transformation vector and the lower panels show bright field images where nodules are present on the mutant roots complemented by the WT RPG protein. Scale bars: 5 mm.
(TIF)

S3 Fig Map-based cloning of the *rpg-6*. (A) Overview of the region identified by rough mapping on chromosome 5 showing the locations of CERBERUS and RPG. (B) The region was further defined by fine mapping and the mutation identified in *RPG* (red asterisk). (C) Overview of the *RPG* genomic region showing the location of the *rpg-1*, *rpg-2* and *rpg-6* mutations.
(TIF)

S4 Fig Infection and nodulation phenotypes of *rpg LORE1* insertion mutants. (A) Number of infection events in wild type (WT) (n = 8, 5) and the *rpg-3* (n = 9, 6), *rpg-4* (n = 9, 7) and *rpg-5* (n = 9, 4) *LORE1* insertion mutants. The numbers of infection events per plant were scored 4 and 10 dpi with *M*. *loti* R7A/LacZ. IF, infection foci; ITs, infection threads; ‘others’, abnormal ITs in root hairs. (B) Nodule number was scored in WT (n = 6) and *rpg-3* (n = 12), *rpg-4* (n = 9) and *rpg-5* (n = 8) *LORE1* insertion mutant plants 21 dpi with *M*. *loti* R7A/LacZ. Asterisks indicate significant differences (Students *t*-test), between WT and *rpg* mutants at the indicated time points. (C-D) Typical infection and nodule phenotypes of WT and *rpg LORE1* insertion mutants stained with X-Gal 10 days after inoculation with *M*. *loti* R7A/LacZ. (E) Sections of nodules formed on WT and the *rpg-3*, *rpg-4* and *rpg-5 LORE1* insertion mutants. Roots were inoculated with *M*. *loti* R7A and nodules were sectioned and stained with toluidine blue two weeks after inoculation. Scale bars: 20 μm (C); 100 μm (D) and 100 μm (E).
(TIF)

S5 Fig Nodule phenotype and complementation of *rpg-6*. (A) Images of a WT nodulated root (left) and a root with uninfected nodule primordia from the *rpg-6* mutant (right) 14 dpi with *M*. *loti* MAFF303099/DsRED. The upper images were taken using white light, the central images show DsRed fluorescence and the bottom images are overlays of the other two images. (B) Mean number of infected nodules and uninfected nodule primordia observed on WT (n = 8) and *rpg-6* mutants (n = 12) at 14 dpi. Asterisks indicate the significant difference (Students *t*-test) between WT and *rpg-6*. Scale bars: 1 mm (A).
(TIF)

S6 Fig *RPG* expression in *LORE1* insertion mutant roots after inoculation with *M*. *loti* R7A. (A) Structure of *RPG*, showing the location of the *rpg* mutations and the locations of the primers used for qRT-PCR. (B) qRT-PCR analysis of *RPG* transcript levels in wild type (WT) and *rpg* mutant roots. Roots were harvested at 10 dpi. Expression is relative to that of WT and normalized to the *L*. *japonicus Ubiquitin* transcript levels. Asterisks indicate significant differences (Students *t*-test) between WT and *rpg* mutants.
(TIF)

S7 Fig Expression of early nodulation genes in wild type (WT) and *rpg* mutant roots after inoculation with *M*. *loti* R7A. (A-D) qRT-PCR was used to measure expression levels of *NIN*, *NPL*, *RINRK1* and *VPY1* in roots of WT, *rpg-1* and *rpg-2* mutants. Plants were grown on FP agar and were assayed five days after mock inoculation or inoculation with *M*. *loti* R7A. Expression is relative to that of mock-treated samples and normalized to *L*. *japonicus Ubiquitin* transcript levels. Asterisks indicate significant difference (Students *t*-test), between inoculated and mock-inoculated WT.
(TIF)

S8 Fig Root colonization of *rpg* mutants by the AM fungus *R*. *irregularis*. (A) Quantitative AM colonization of WT (n = 11), *rpg-1* (n = 12) and *rpg-2* (n = 13) mutants assayed by ink-vinegar staining to visualize fungal by light microscopy at five weeks after inoculation. Frequency of root colonization (total structures, arbuscules [Arb%] and hyphae [Hyp%] were determined with the modified grid-line intersection method. (B) AM colonization of WT, *rpg-1*, and *rpg-2* plants. Five weeks after inoculation with AMF, roots were stained with Alexa Fluora 488 wheat germ-agglutinin (WGA) and photographed using a confocal-laser scanning microscope. Scale bars: 20 μm.
(TIF)

S9 Fig GFP-RPG can rescue *rpg-1* phenotype. (A -B) Quantification of infection events and nodule numbers in *rpg-1* or stably transformed with p*35S*:*GFP-RPG*. The average numbers of infection events and nodule numbers per plant was scored 7 and 14 dpi with *M*. *loti* R7A/LacZ, respectively (n>8). Asterisks indicate significant differences (Students *t*-test), between *rpg-1* and the rescued line. (C) Infection threads (7 dpi) and nodule phenotype (14 dpi) of *rpg-1* and GFP-RPG/*rpg-1* stable transgenic line inoculated with *M*. *loti* R7A/LacZ. Scale bars: 25 μm (root hair) and 5mm (root).
(TIF)

S10 Fig Nodulation phenotype of the *rpg-1* mutant stably transformed with p*RPG*:*GFP-RPG*. (A-B) Quantification of infection events and nodules in wild type (WT), *rpg-1*, and *rpg-1* stably transformed with p*RPG*:*GFP-RPG*. The average numbers of infection events and nodule numbers per plant was scored 7 and 14 dpi with *M*. *loti* R7A/LacZ, respectively (n>11). Asterisks indicate significant differences (Students *t*-test), between mutant lines and WT. (C) Infection threads and nodule phenotype of WT and stable transgenic line 7 dpi with *M*. *loti* R7A/LacZ. Scale bars: 25 μm (root hair) and 100 μm (nodule).
(TIF)

S11 Fig Western blot analysis of full-length RPG and domains of RPG fused to GFP extracted from transiently transformed *N*. *benthamiana* leaves. *L*. *japonicus Ubiquitin* promoter driven *GFP-RPG*, *GFP-NT-C2*, or *GFP-CC*, and the p*35S*: *Myc-GFP* in *A*. *tumefaciens* were introduced into *N*. *benthamiana* leaves. Two days after agroinfiltration proteins were extracted and the GFP fusion proteins were enriched with anti-GFP Affinity beads 4FF. After SDS-PAGE immunoblots were analyzed using anti-GFP antibodies.
(TIF)

S12 Fig RPG CC can rescue *rpg-1* nodulation phenotype. Roots of *L*. *japonicus* WT and *rpg-1* mutant were transformed with the vector control (EV), or with constructs encoding intact RPG (p*UB*:*RPG*), its NT-C2 domain (p*UB*:*NT-C2*) *or* RPG lacking the NT-C2 domain (p*UB*:*CC*). (A) Nodules were imaged three weeks after inoculation with *M*. *loti* R7A/LacZ. The upper panels are epifluorescence microscopy images showing GFP expression and the lower panels show bright field images. (B) The nodule numbers were scored three weeks after inoculation with *M*. *loti* R7A/LacZ (n>14). Asterisks indicate significant differences (Students *t*-test), between *rpg-1* lines and WT/ EV. Scale bars: 2 mm.
(TIF)

S13 Fig Co-localization of RPG with CERBERUS. (A-B) GFP-RPG (green) and CERBERUS-mCherry (magenta) were co-expressed in *N*. *benthamiana* leaf cells (A) or in *L*. *japonicus* root protoplasts (B) using a DNA-PEG-calcium transfection method. Plots (A‴) and (B‴) show fluorescence intensities of GFP-RPG and CERBERUS-mCherry in regions of interest (indicated by white line in [A″] and [B″]). Scale bars: 25 μm (A); 10 μm (B).
(TIF)

S14 Fig Co-localization of RPG with ER and TGN/EE markers but not Golgi or MVB markers. Protein subcellular localizations were analyzed by confocal microscopy of proteins co-expressed in *N*. *benthamiana* leaf cells. (A) Fluorescence from GFP-RPG (green) and (A′) the ER marker Sec12-PHB-mCherry (magenta) was imaged and the merged image (A″) shows that the punctate expression of GFP-RPG occurs in specific regions that are associated with fluorescence from Sec12-PHB-mCherry. A plot (A‴) of fluorescence intensities of GFP-RPG and Sec12-PHB-mCherry fluorescence in a region of interest (white line in A″) shows that GFP-RPG colocalized with Sec12-PHB-mCherry, whereas other regions of Sec12-PHB-mCherry do not show GFP-RPG localization. (B) Fluorescence from the TGN/EE marker HAP-13-GFP (green) and (B′) from mCherry-RPG colocalized based on the merged image (B″) and (B‴) the plot of fluorescence intensities of HAP-13-GFP and mCherry-RPG in the area of interest (marked with a line in B″). (C) Fluorescence from GFP-RPG and (C′) the MVB marker ARA6-mCherry. The punctate expression of GFP-RPG (green) and the punctate localization of foci of the multivesicular body (MVB) marker ARA6-mCherry SR2 did not colocalize based on the merged image (C″) and the enlargement of it (C‴). (D) The green GFP-RPG fluorescence and (D′) red fluorescence from mRFP fused to the-vascular sorting peptide 2 (mRFP-VSR2) did not colocalize based on the merged image (D″) and the enlargement of it (D‴). (E) The fluorescence from the Golgi marker CD3-963-GFP (green) and (E′) mCherry-RPG (magenta) did not colocalize based on the merged image (E″) and the enlargement of it (E‴). (F) The RPG-CERBERUS BiFC construct (yellow) and (F′) the ER marker Sec12-PHB-mCherry (magenta) colocalized based on the merged image (F″) and the Plot (F‴) showing fluorescence intensities of Venus and Sec12-PHB-mCherry in regions of interest (white line in F″). Scale bars: 25 μm (A-F).
(TIF)

S1 Table Primers used in this study. (XLSX)

S2 Table Constructs used in this study. (XLSX)

S1 Data All numerical for quantitation figures. (XLSX)
