# Supplementary figures and images for: RPG interacts with E3-ligase CERBERUS to mediate rhizobial infection in Lotus japonicus
Source: PLoS Genet. 2023 Feb 3;19(2):e1010621. doi: 10.1371/journal.pgen.1010621 (PMC9931111; doi:10.1371/journal.pgen.1010621)

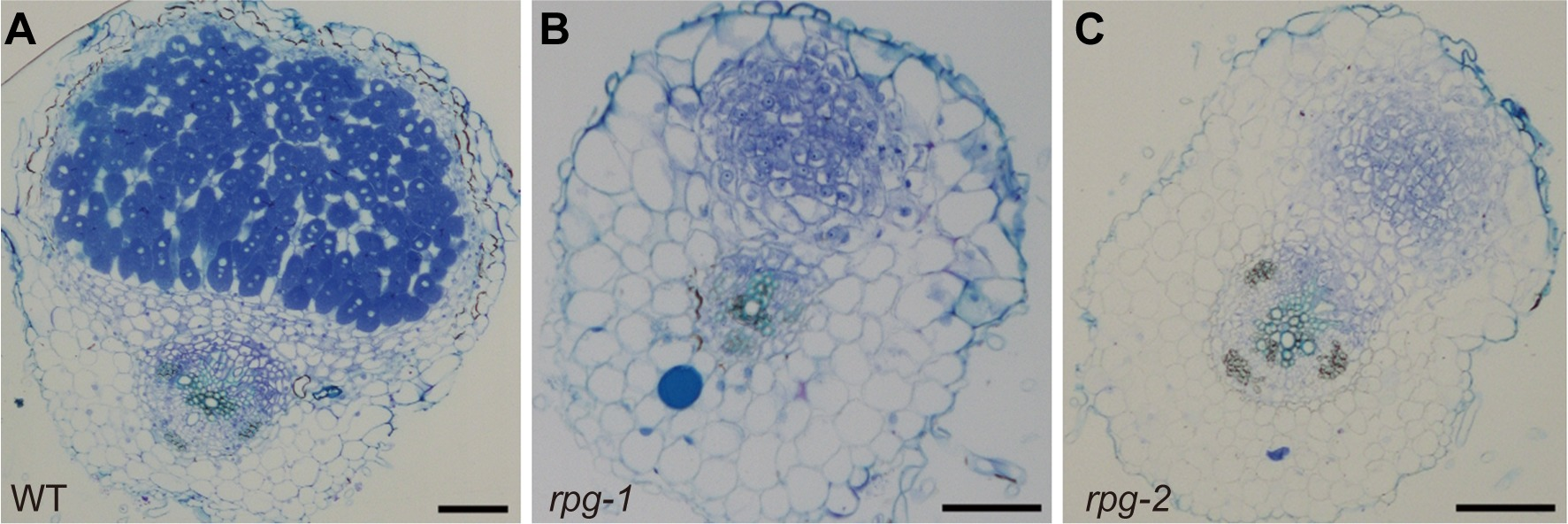

Supplement: S1 Fig — (A-C) Section of 3-week old nodules on wild type plants were well colonization, but no colonization was observed in rpg-1 and rpg-2. Scale bars: 100 μm. (TIF) [file pgen.1010621.s001.tif]

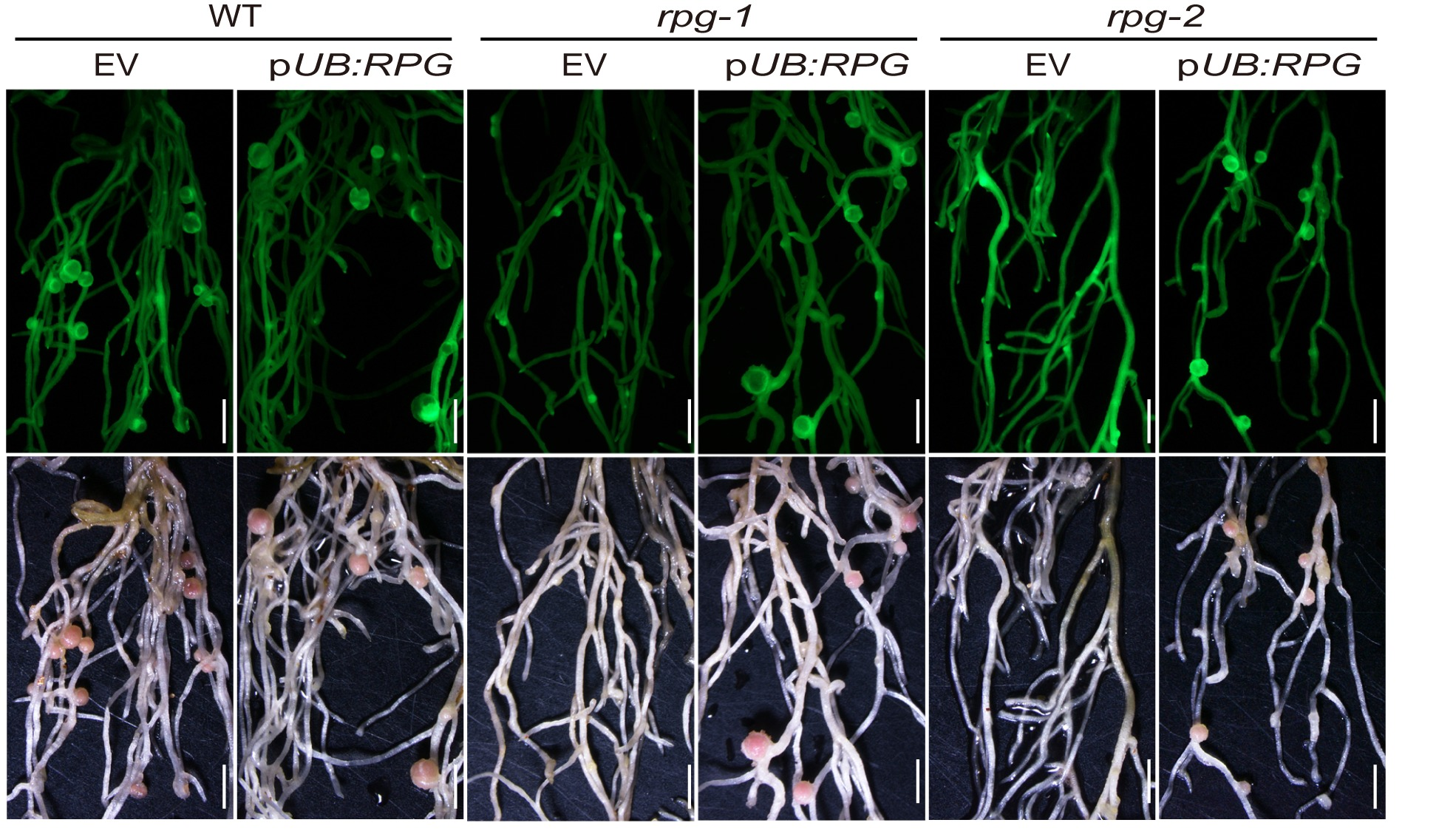

Supplement: S2 Fig — Representative transgenic hairy roots of L. japonicus WT, rpg-1 and rpg-2 plants transformed with the empty vector control (EV) or pUB:RPG three weeks after inoculation with M. loti R7A/LacZ. The upper panels are epifluorescence microscopy images showing GFP expression from the transformation vector and the lower panels show bright field images where nodules are present on the mutant roots complemented by the WT RPG protein. Scale bars: 5 mm. (TIF) [file pgen.1010621.s002.tif]

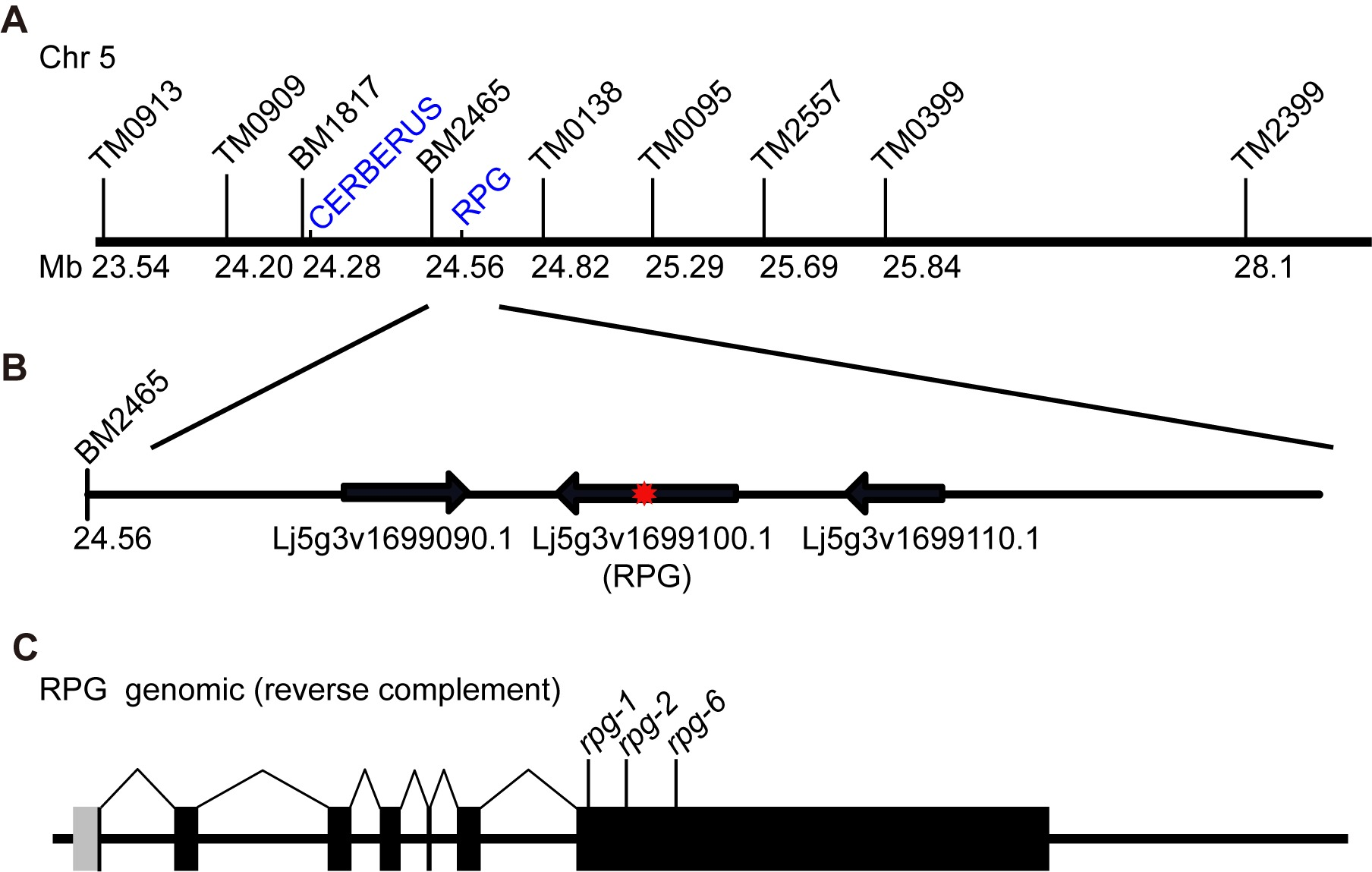

Supplement: S3 Fig — (A) Overview of the region identified by rough mapping on chromosome 5 showing the locations of CERBERUS and RPG. (B) The region was further defined by fine mapping and the mutation identified in RPG (red asterisk). (C) Overview of the RPG genomic region showing the location of the rpg-1, rpg-2 and rpg-6 mutations. (TIF) [file pgen.1010621.s003.tif]

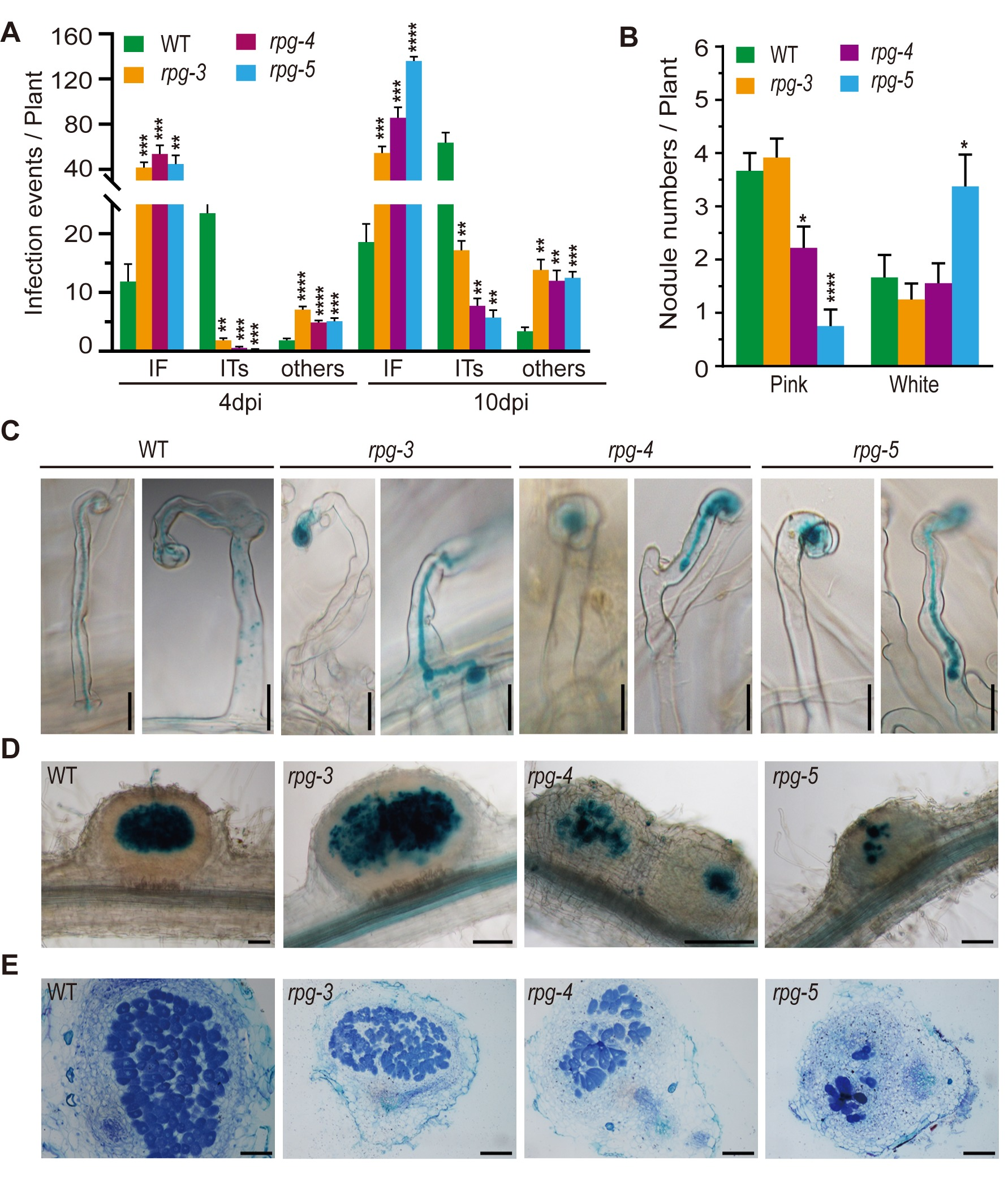

Supplement: S4 Fig — (A) Number of infection events in wild type (WT) (n = 8, 5) and the rpg-3 (n = 9, 6), rpg-4 (n = 9, 7) and rpg-5 (n = 9, 4) LORE1 insertion mutants. The numbers of infection events per plant were scored 4 and 10 dpi with M. loti R7A/LacZ. IF, infection foci; ITs, infection threads; ‘others’, abnormal ITs in root hairs. (B) Nodule number was scored in WT (n = 6) and rpg-3 (n = 12), rpg-4 (n = 9) and rpg-5 (n = 8) LORE1 insertion mutant plants 21 dpi with M. loti R7A/LacZ. Asterisks indicate significant differences (Students t-test), between WT and rpg mutants at the indicated time points. (C-D) Typical infection and nodule phenotypes of WT and rpg LORE1 insertion mutants stained with X-Gal 10 days after inoculation with M. loti R7A/LacZ. (E) Sections of nodules formed on WT and the rpg-3, rpg-4 and rpg-5 LORE1 insertion mutants. Roots were inoculated with M. loti R7A and nodules were sectioned and stained with toluidine blue two weeks after inoculation. Scale bars: 20 μm (C); 100 μm (D) and 100 μm (E). (TIF) [file pgen.1010621.s004.tif]

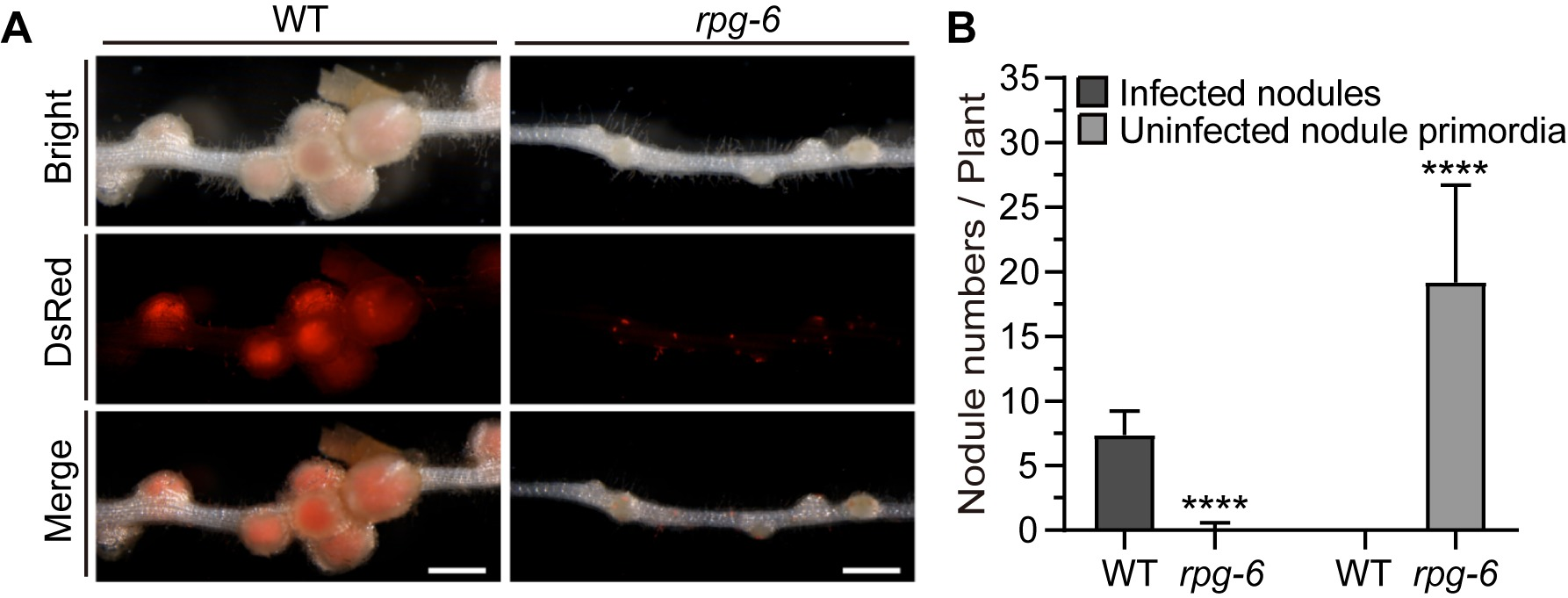

Supplement: S5 Fig — (A) Images of a WT nodulated root (left) and a root with uninfected nodule primordia from the rpg-6 mutant (right) 14 dpi with M. loti MAFF303099/DsRED. The upper images were taken using white light, the central images show DsRed fluorescence and the bottom images are overlays of the other two images. (B) Mean number of infected nodules and uninfected nodule primordia observed on WT (n = 8) and rpg-6 mutants (n = 12) at 14 dpi. Asterisks indicate the significant difference (Students t-test) between WT and rpg-6. Scale bars: 1 mm (A). (TIF) [file pgen.1010621.s005.tif]

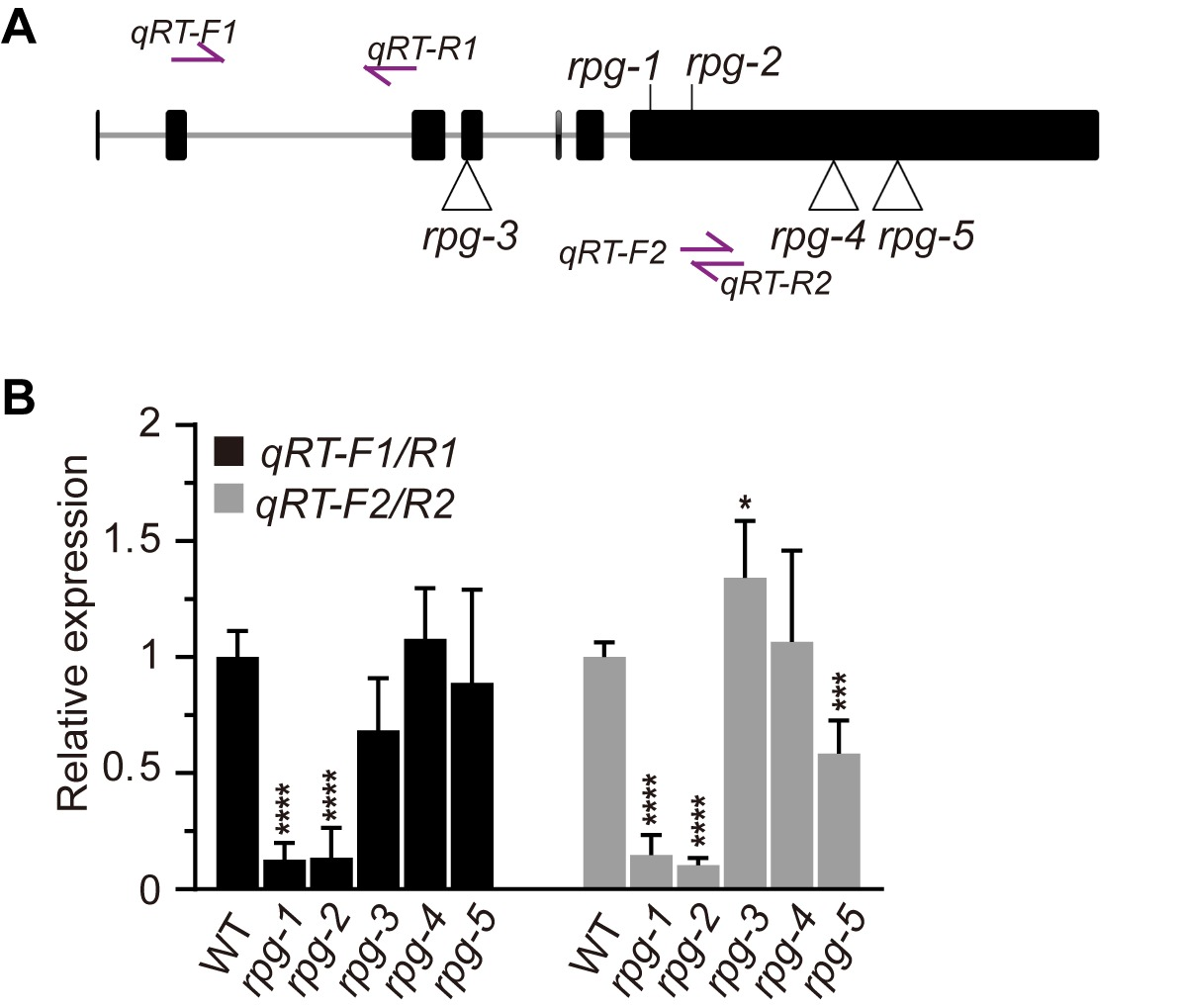

Supplement: S6 Fig — (A) Structure of RPG, showing the location of the rpg mutations and the locations of the primers used for qRT-PCR. (B) qRT-PCR analysis of RPG transcript levels in wild type (WT) and rpg mutant roots. Roots were harvested at 10 dpi. Expression is relative to that of WT and normalized to the L. japonicus Ubiquitin transcript levels. Asterisks indicate significant differences (Students t-test) between WT and rpg mutants. (TIF) [file pgen.1010621.s006.tif]

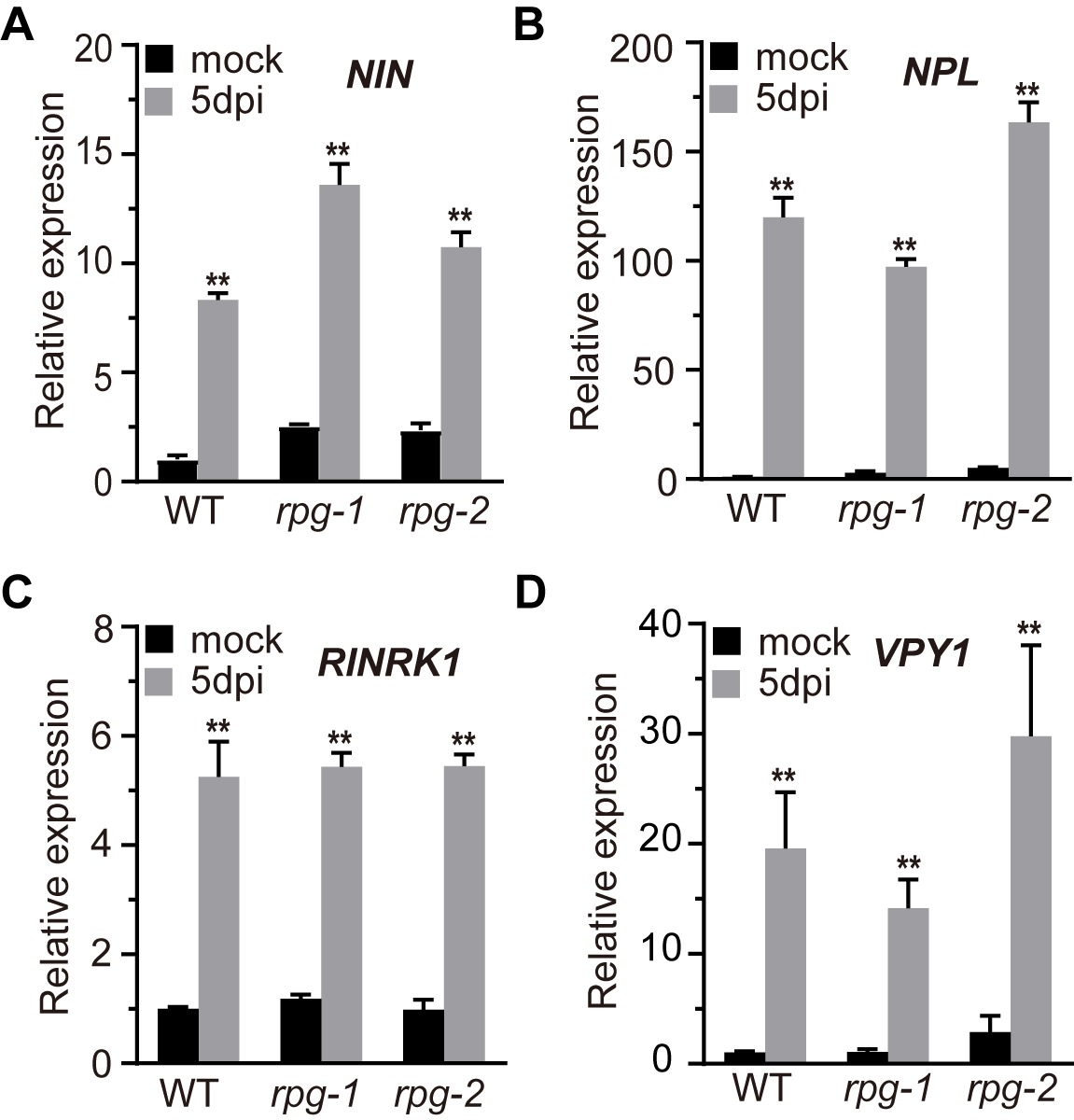

Supplement: S7 Fig — (A-D) qRT-PCR was used to measure expression levels of NIN, NPL, RINRK1 and VPY1 in roots of WT, rpg-1 and rpg-2 mutants. Plants were grown on FP agar and were assayed five days after mock inoculation or inoculation with M. loti R7A. Expression is relative to that of mock-treated samples and normalized to L. japonicus Ubiquitin transcript levels. Asterisks indicate significant difference (Students t-test), between inoculated and mock-inoculated WT. (TIF) [file pgen.1010621.s007.tif]

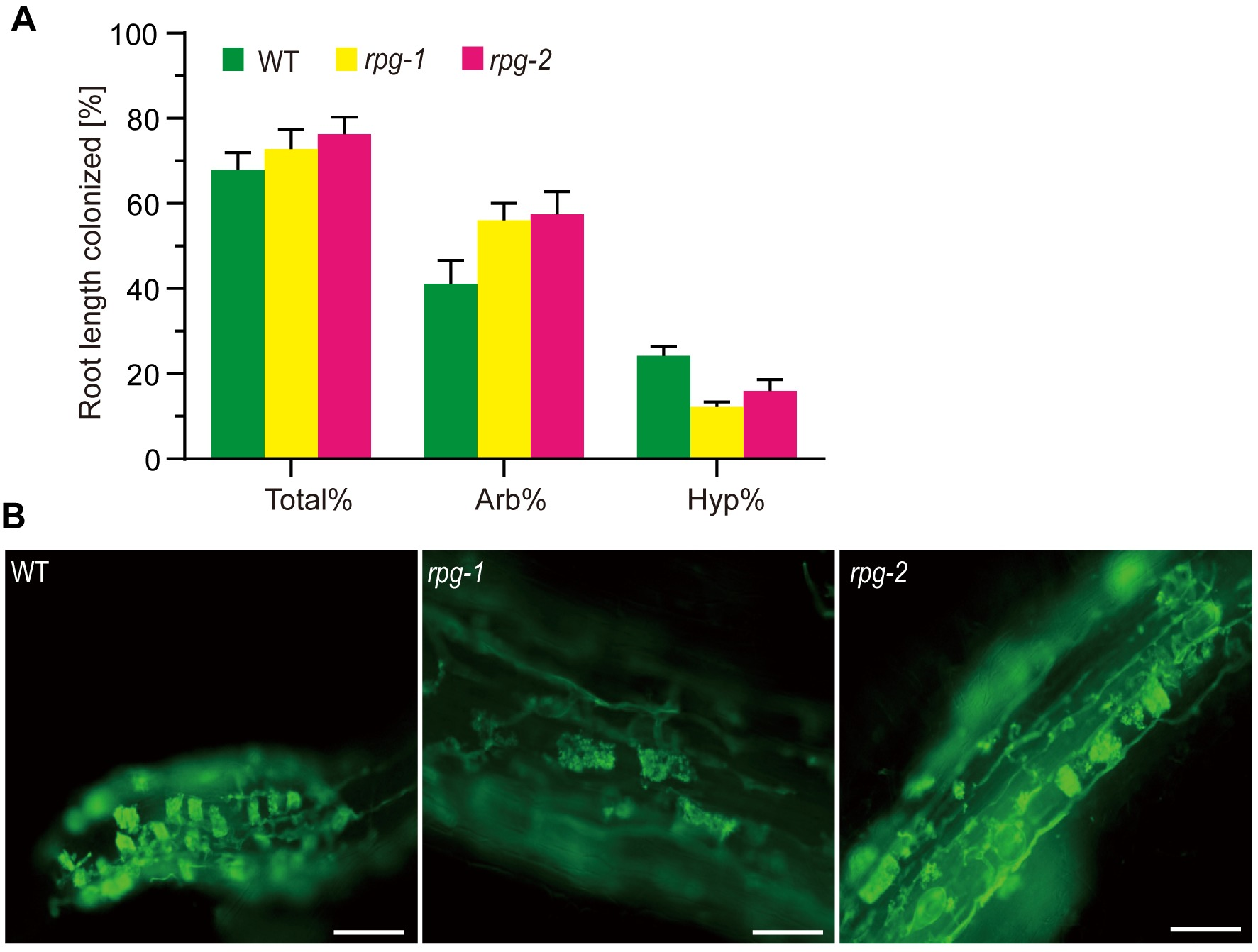

Supplement: S8 Fig — (A) Quantitative AM colonization of WT (n = 11), rpg-1 (n = 12) and rpg-2 (n = 13) mutants assayed by ink-vinegar staining to visualize fungal by light microscopy at five weeks after inoculation. Frequency of root colonization (total structures, arbuscules [Arb%] and hyphae [Hyp%] were determined with the modified grid-line intersection method. (B) AM colonization of WT, rpg-1, and rpg-2 plants. Five weeks after inoculation with AMF, roots were stained with Alexa Fluora 488 wheat germ-agglutinin (WGA) and photographed using a confocal-laser scanning microscope. Scale bars: 20 μm. (TIF) [file pgen.1010621.s008.tif]

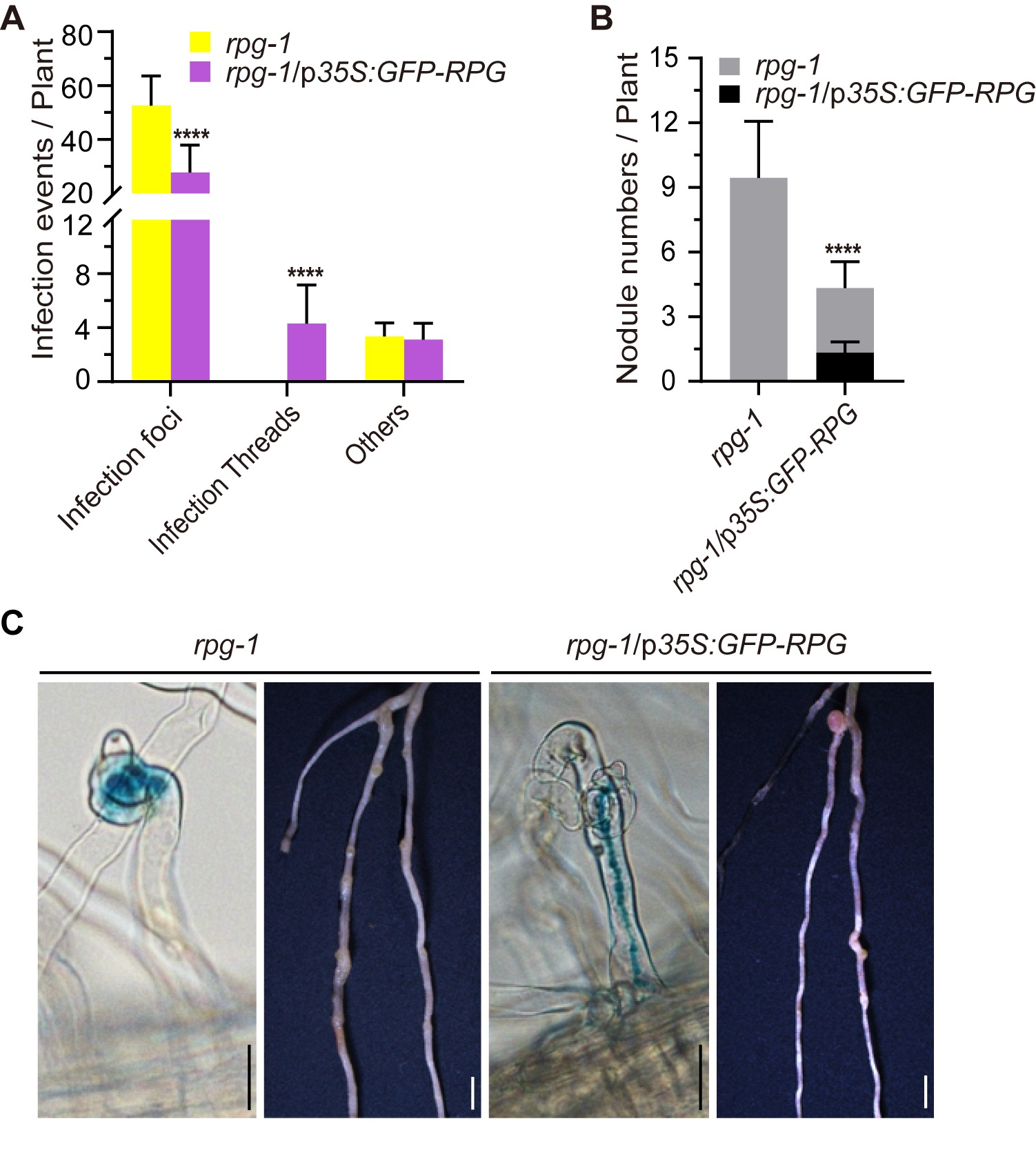

Supplement: S9 Fig — (A -B) Quantification of infection events and nodule numbers in rpg-1 or stably transformed with p35S:GFP-RPG. The average numbers of infection events and nodule numbers per plant was scored 7 and 14 dpi with M. loti R7A/LacZ, respectively (n>8). Asterisks indicate significant differences (Students t-test), between rpg-1 and the rescued line. (C) Infection threads (7 dpi) and nodule phenotype (14 dpi) of rpg-1 and GFP-RPG/rpg-1 stable transgenic line inoculated with M. loti R7A/LacZ. Scale bars: 25 μm (root hair) and 5mm (root). (TIF) [file pgen.1010621.s009.tif]

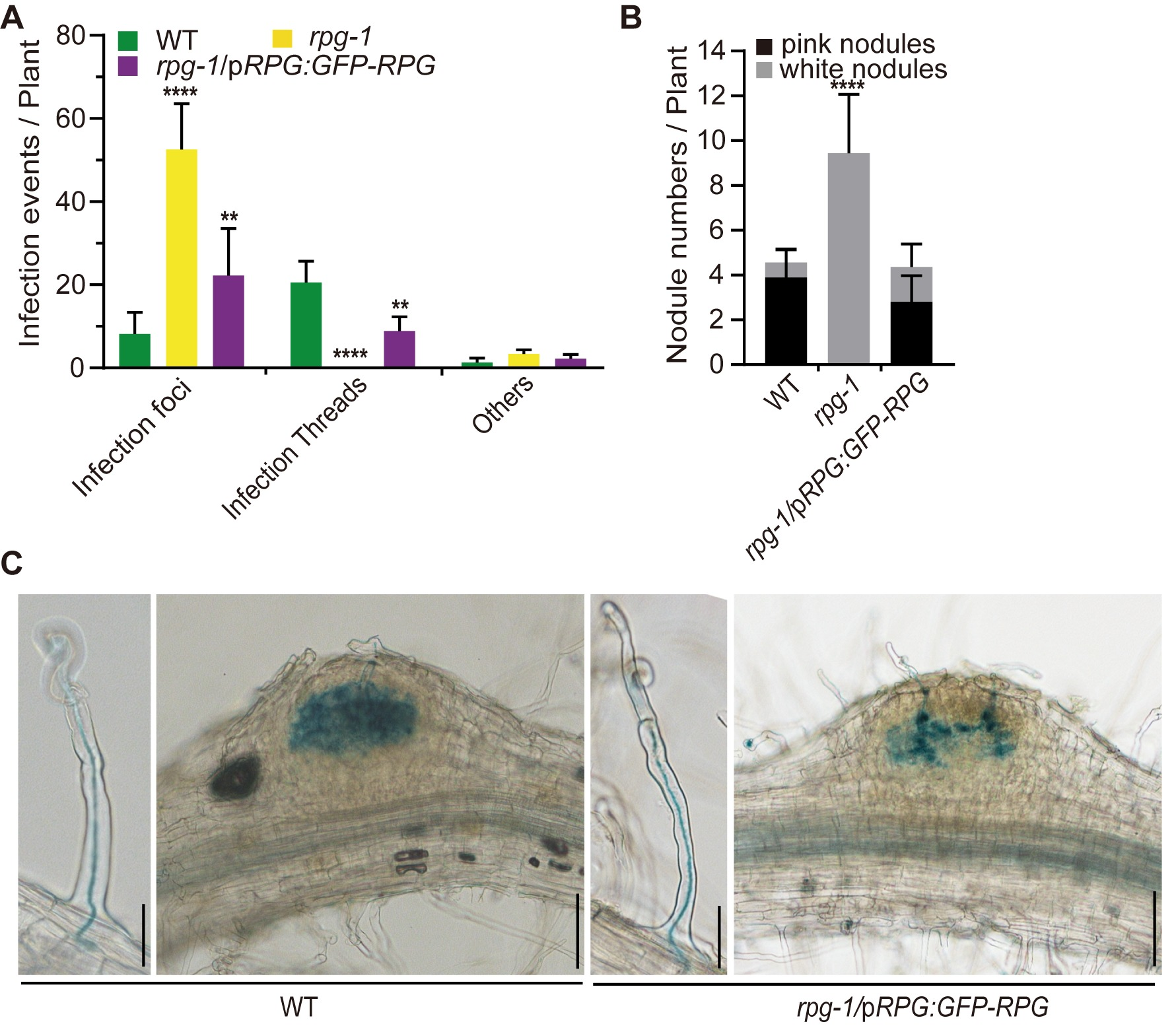

Supplement: S10 Fig — (A-B) Quantification of infection events and nodules in wild type (WT), rpg-1, and rpg-1 stably transformed with pRPG:GFP-RPG. The average numbers of infection events and nodule numbers per plant was scored 7 and 14 dpi with M. loti R7A/LacZ, respectively (n>11). Asterisks indicate significant differences (Students t-test), between mutant lines and WT. (C) Infection threads and nodule phenotype of WT and stable transgenic line 7 dpi with M. loti R7A/LacZ. Scale bars: 25 μm (root hair) and 100 μm (nodule). (TIF) [file pgen.1010621.s010.tif]

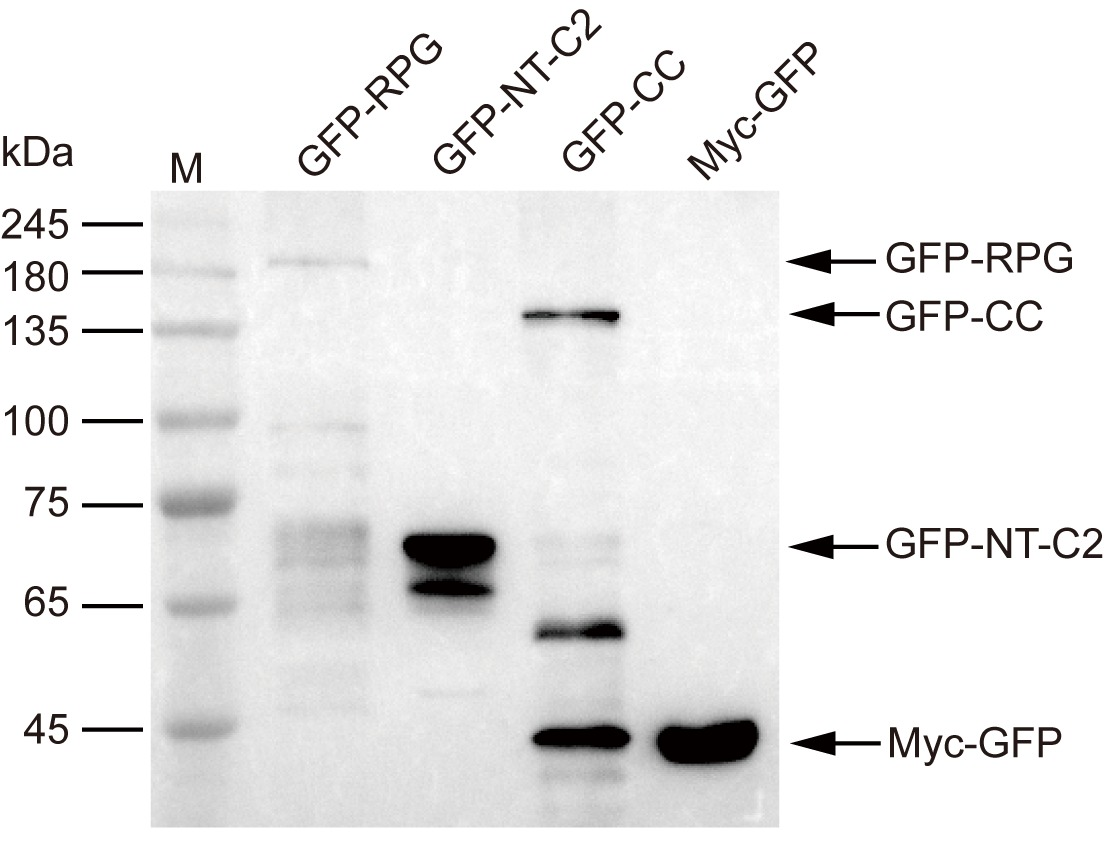

Supplement: S11 Fig — L. japonicus Ubiquitin promoter driven GFP-RPG, GFP-NT-C2, or GFP-CC, and the p35S: Myc-GFP in A. tumefaciens were introduced into N. benthamiana leaves. Two days after agroinfiltration proteins were extracted and the GFP fusion proteins were enriched with anti-GFP Affinity beads 4FF. After SDS-PAGE immunoblots were analyzed using anti-GFP antibodies. (TIF) [file pgen.1010621.s011.tif]

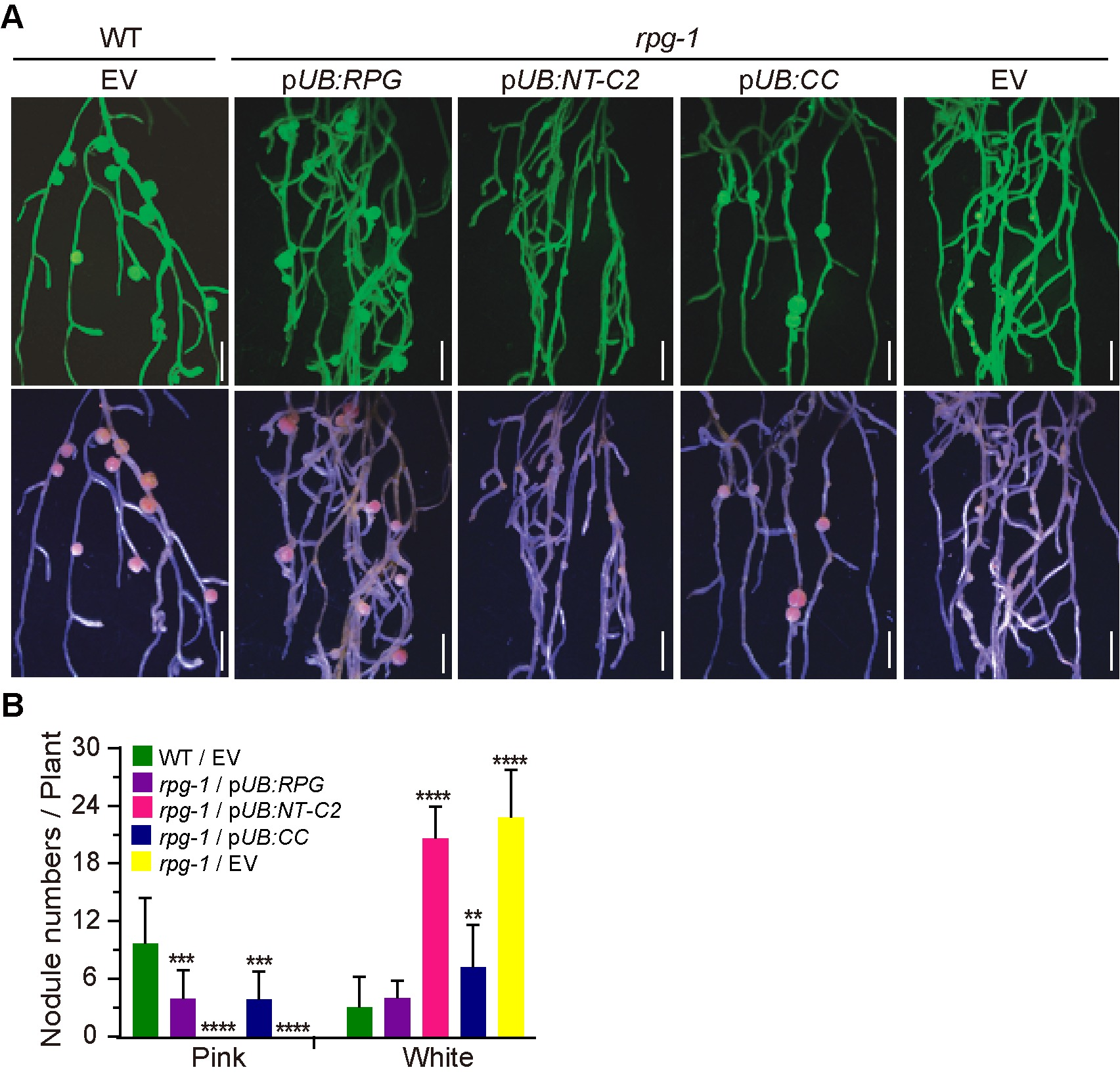

Supplement: S12 Fig — Roots of L. japonicus WT and rpg-1 mutant were transformed with the vector control (EV), or with constructs encoding intact RPG (pUB:RPG), its NT-C2 domain (pUB:NT-C2) or RPG lacking the NT-C2 domain (pUB:CC). (A) Nodules were imaged three weeks after inoculation with M. loti R7A/LacZ. The upper panels are epifluorescence microscopy images showing GFP expression and the lower panels show bright field images. (B) The nodule numbers were scored three weeks after inoculation with M. loti R7A/LacZ (n>14). Asterisks indicate significant differences (Students t-test), between rpg-1 lines and WT/ EV. Scale bars: 2 mm. (TIF) [file pgen.1010621.s012.tif]

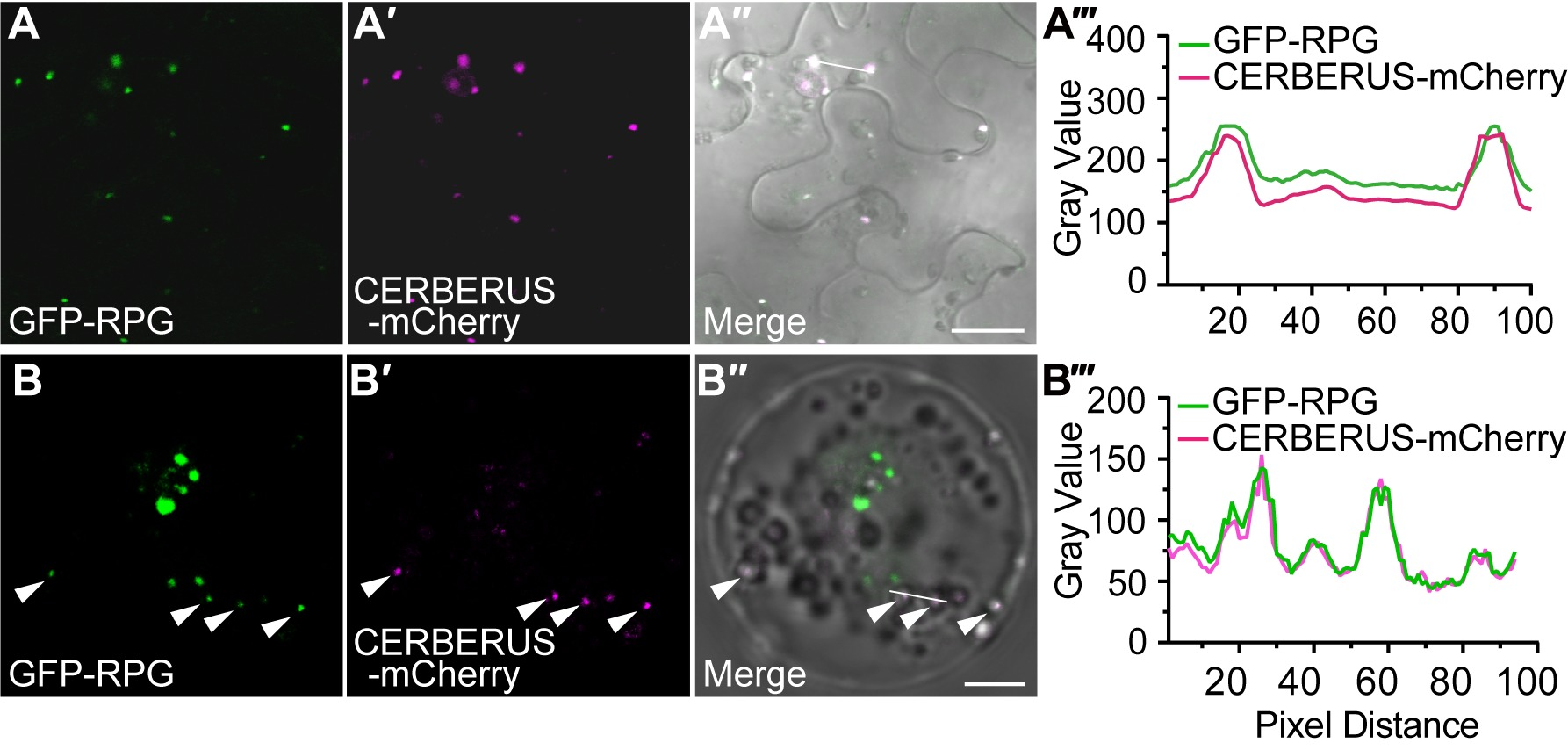

Supplement: S13 Fig — (A-B) GFP-RPG (green) and CERBERUS-mCherry (magenta) were co-expressed in N. benthamiana leaf cells (A) or in L. japonicus root protoplasts (B) using a DNA-PEG-calcium transfection method. Plots (A‴) and (B‴) show fluorescence intensities of GFP-RPG and CERBERUS-mCherry in regions of interest (indicated by white line in [A″] and [B″]). Scale bars: 25 μm (A); 10 μm (B). (TIF) [file pgen.1010621.s013.tif]

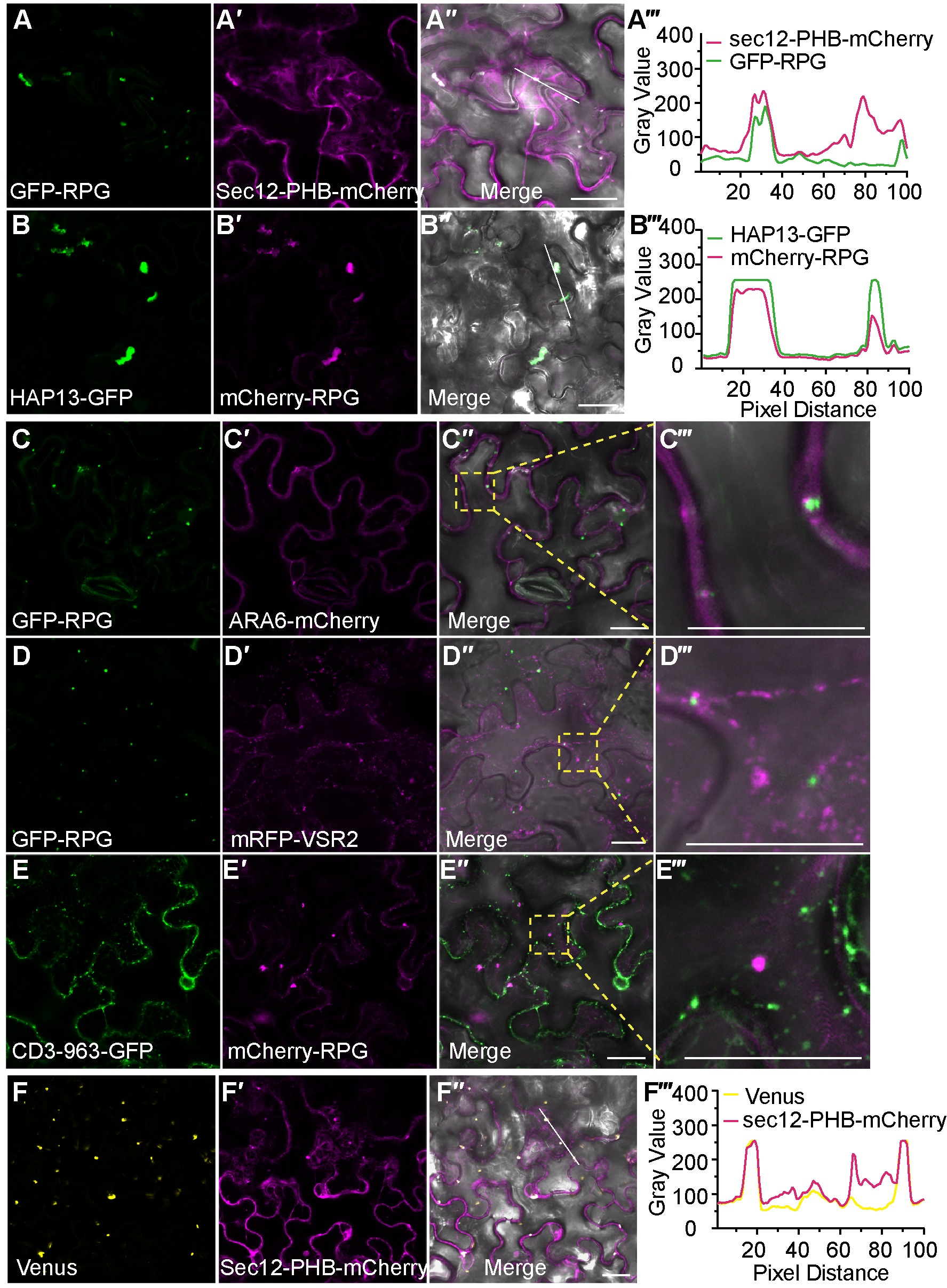

Supplement: S14 Fig — Protein subcellular localizations were analyzed by confocal microscopy of proteins co-expressed in N. benthamiana leaf cells. (A) Fluorescence from GFP-RPG (green) and (A′) the ER marker Sec12-PHB-mCherry (magenta) was imaged and the merged image (A″) shows that the punctate expression of GFP-RPG occurs in specific regions that are associated with fluorescence from Sec12-PHB-mCherry. A plot (A‴) of fluorescence intensities of GFP-RPG and Sec12-PHB-mCherry fluorescence in a region of interest (white line in A″) shows that GFP-RPG colocalized with Sec12-PHB-mCherry, whereas other regions of Sec12-PHB-mCherry do not show GFP-RPG localization. (B) Fluorescence from the TGN/EE marker HAP-13-GFP (green) and (B′) from mCherry-RPG colocalized based on the merged image (B″) and (B‴) the plot of fluorescence intensities of HAP-13-GFP and mCherry-RPG in the area of interest (marked with a line in B″). (C) Fluorescence from GFP-RPG and (C′) the MVB marker ARA6-mCherry. The punctate expression of GFP-RPG (green) and the punctate localization of foci of the multivesicular body (MVB) marker ARA6-mCherry SR2 did not colocalize based on the merged image (C″) and the enlargement of it (C‴). (D) The green GFP-RPG fluorescence and (D′) red fluorescence from mRFP fused to the-vascular sorting peptide 2 (mRFP-VSR2) did not colocalize based on the merged image (D″) and the enlargement of it (D‴). (E) The fluorescence from the Golgi marker CD3-963-GFP (green) and (E′) mCherry-RPG (magenta) did not colocalize based on the merged image (E″) and the enlargement of it (E‴). (F) The RPG-CERBERUS BiFC construct (yellow) and (F′) the ER marker Sec12-PHB-mCherry (magenta) colocalized based on the merged image (F″) and the Plot (F‴) showing fluorescence intensities of Venus and Sec12-PHB-mCherry in regions of interest (white line in F″). Scale bars: 25 μm (A-F). (TIF) [file pgen.1010621.s014.tif]
